# Punishing the privileged: Selfish offers from high-status allocators elicit greater punishment from third-party arbitrators

**DOI:** 10.1371/journal.pone.0232369

**Published:** 2020-05-14

**Authors:** Bradley D. Mattan, Denise M. Barth, Alexandra Thompson, Oriel FeldmanHall, Jasmin Cloutier, Jennifer T. Kubota

**Affiliations:** 1 Annenberg School for Communication, University of Pennsylvania, Philadelphia, PA, United States of America; 2 Department of Psychological and Brain Sciences, University of Delaware, Newark, DE, United States of America; 3 College of Liberal and Professional Studies, University of Pennsylvania, Philadelphia, PA, United States of America; 4 Department of Cognitive, Linguistic, and Psychological Sciences, Brown University, Providence, RI, United States of America; 5 Department of Political Science and International Relations, University of Delaware, Newark, DE, United States of America; Middlesex University, UNITED KINGDOM

## Abstract

Individuals high in socioeconomic status (SES) are often viewed as valuable members of society. However, the appeal of high-SES people exists in tension with our aversion to inequity. Little experimental work has directly examined how people rectify inequitable distributions between two individuals varying in SES. The objective of the present study was to examine how disinterested third parties adjudicate inequity in the context of concrete financial allocations between a selfish allocator and a recipient who was the victim of the allocator’s selfish offer. Specifically, this study focused on whether knowing the SES of the victim or the allocator affected the participant’s decisions to punish the selfish allocator. In two experiments (*N* = 999), participants completed a modified third-party Ultimatum Game in which they arbitrated inequitable exchanges between an allocator and a recipient. Although participants generally preferred to redistribute inequitable exchanges without punishing players who made unfair allocations, we observed an increased preference for punitive solutions as offers became increasingly selfish. This tendency was especially pronounced when the victim was low in SES or when the perpetrator was high in SES, suggesting a tendency to favor the disadvantaged even among participants reporting high subjective SES. Finally, punitive responses were especially likely when the context emphasized the allocator’s privileged status rather than the recipient’s underprivileged status. These findings inform our understanding of how SES biases retributive justice even in non-judicial contexts that minimize the salience of punishment.

## Introduction

The principle of equity is celebrated as a universal human value [1–3]. Yet even in countries that prize equality, there are still observed preferences for some degree of inequity [4–10]. This may be because the privileged have historically promoted the idea that hierarchy is established through non-exploitative means such as talent, hard work, or appointment from higher authority [11,12]. However, when the privileged are exploitative [13] or even insufficiently generous [14,15], people often desire sanctions for these privileged perpetrators. For example, in 2018, the former extremely wealthy hedge fund manager and pharmaceutical CEO Martin Shkreli was convicted and sentenced to seven years in prison for defrauding hedge fund investors who trusted him with their money [16]. Beyond Shkreli’s patently odious persona [17], prospective jury members seemed more concerned with Shkreli’s exploitation of the disadvantaged (viz., people who depend on affordable prescription drugs) rather than the privileged hedge fund investors he illegally defrauded [18]. Shkreli’s public vilification for victimizing the disadvantaged highlights the growing public concern about the treatment of the “haves” and “have nots” [19,20] and raises the question of how the socioeconomic status (SES) of victims and perpetrators of inequity shapes the way third parties (e.g., judges, juries, court of public opinion) decide how to correct injustice. In particular, how does a perpetrator’s or victim’s SES shape punishment preferences when non-punitive solutions are also available [21]? To address these questions, the current study examined whether and how impartial third parties act to correct unfair distributions between individuals varying in SES. Based on previous work [21,22], we anticipated that participants would generally prefer non-punitive over punitive solutions, but that SES would nonetheless influence a third party’s willingness to punish. We predicted an increased preference for punitive solutions for highly unjust allocations if they were perpetrated against the disadvantaged (i.e., low-SES individuals: Experiment 1) or committed by the privileged (i.e., high-SES individuals: Experiment 2). We also explored how the perceiver’s SES impacted these preferences for punitive solutions to financial inequity.

Research shows that third parties are willing to punish perpetrators of inequity even when this might come at a personal cost [1–3,23–25]. It has been argued that this sensitivity to fairness emerged relatively recently in our evolutionary history as human societies have grown in complexity [26], developing in tension with our heritage as a hierarchy-sensitive species [27]. However, even non-human primates accustomed to relatively steep dominance hierarchies (e.g., macaques, chimpanzees) are sensitive to and take action to mitigate inequality [28]. Given that social hierarchies are an intrinsic and systemic part of human culture [29] and organizations [30]—which help to shape our motives [30,31], affect [32,33], self-presentation strategies [34–36], evaluations of others [37–39], and support for redistributive socioeconomic policies [40–43]—it is critical to examine how inequity is perceived and addressed in social hierarchies. In the real world, systemic inequalities stemming from race and poverty [43–45] form the backdrop against which many fairness judgments are determined. Although several decades of research have examined fairness and punishment in the context of stratified social hierarchies [13,46–51], these studies typically focus on the participant’s own status in that hierarchy [e.g., 47–49]. Some research has focused on punishment decisions for people of varying social status levels [e.g., 13,46,50,51]. However, these studies have tended to focus specifically on criminal offenders in an explicitly judicial context, showing that high-status perpetrators are seen as more intentional and/or callous, therefore deserving greater punishment than their lower status counterparts [13,46]. This preference for punishing selfishness among the rich is further enhanced among those who generally desire greater equity [50].

Building on this literature, this is the first experimental investigation to our knowledge that directly manipulates the perceived status of perpetrators or victims in a context that minimizes the emphasis on criminality. This is important because such judicial contexts may predispose people to think in terms of punishment. Moreover, the degree to which the person’s appearance or attributes reflect a particular criminal stereotype has been shown to influence punishment in judicial contexts [52,53]. Accordingly, we tested whether and how participants acting as impartial third parties would rectify inequity in relatively low-stakes exchanges between partners varying in socioeconomic status (SES). Understanding whether and how inequity is punished in the absence of ascribed criminal behavior and when non-punitive means of correction are available [21,22] provides insight into baseline preferences for punishment for inequity on the basis of SES. Ultimately, this insight could then be applied to numerous real-world contexts involving sanctions for fairness violations. For example, SES and its proxies such as race [54–56] influence real-world sanctions across a wide range of contexts that vary in severity from bureaucratic inconvenience [57] to criminal sentencing [58–64]. Thus, understanding how the victim’s or perpetrator’s SES contributes to fairness and punishment decisions is of paramount importance.

In two experiments (*N* = 999), participants on Amazon Mechanical Turk (MTurk) completed a third-party version of the Ultimatum Game, known as the Justice Game [21,22,65]. Like the original Ultimatum Game, the Justice Game has been shown to capture the desire for punitive solutions to inequity [22,65]. In the Justice Game, participants observed a series of single-shot exchanges between an allocator (Player A) and a recipient (Player B). Each exchange involved a split of funds between the players where Player A always determined how to split the funds with Player B. For example, Player A either acted quite selfishly, giving 20% to Player B and keeping 80%, or only somewhat selfishly, giving 40% to Player B and keeping 60%. Participants played the role of an impartial third party (Player C) and had the power to respond to Player A’s selfish offers in one of three ways: (a) punishment: Player A’s allocation would be reversed such that Player B would end up with the largest share, and Player A would receive the smallest share; (b) compensation: the victim of the selfish offer (i.e., Player B) would receive additional funds to match the amount kept by Player A; and (c) accept: maintain the status quo and accept Player A’s selfish offer. Participants were informed that the results of their decisions would be used to allocate bonuses to Players A and B, who ostensibly were real MTurk workers recruited prior to the experiment (see S1 Text). To make this cover story compelling, every trial involved a split of $1 rather than $10, which would have exceeded the participant’s own compensation and prevailing payment rates on MTurk. Consistent with competing and paradoxical affinities for both fairness and hierarchical order, we posited that participants would (a) tolerate unequal distributions to a degree by preferring non-punitive redistributions, but (b) take strong punitive action against an allocator who perpetrated extreme selfishness [21].

To determine whether exploiting high-SES or low-SES victims is more likely to prompt punitive action, we manipulated the recipient’s (Player B’s but not A’s) ascribed SES as either high or low (see Experiment 1 Methods for details). In line with public opinion about Martin Shkreli [18] and general perceptions of the rich as competent but cold [66,67], we anticipated diminished punishment of allocators who exploit high-SES (vs. low-SES) victims, especially when offers were extremely unfair.

To determine whether high-SES or low-SES perpetrators are more likely to prompt punitive action, we manipulated the allocator’s (Player A’s but not B’s) ascribed SES as either high or low (see Experiment 2 Methods for details). Research suggests that high-status individuals are expected to be more generous or at least non-exploitative toward low-status counterparts [15]. When acting as perpetrators, high-SES perpetrators are seen as more motivated by selfish concerns than low SES perpetrators, which is associated with more severe punishments for rich offenders [46]. Moreover, potentially due to status-based envy of the rich, evidence suggests that people find more pleasure when misfortunes (e.g., punishment) befall the rich versus the poor [68]. We therefore predicted that participants would prefer to punish high-SES allocators, especially when offers were extremely unfair.

## Materials and methods

For all experiments, we have reported all measures, conditions, data exclusions, and sample size determinations, consistent with best practices promoted by the Center for Open Science. Full details are provided in this section and in the online supplemental text. All analyses were conducted in R, version 3.5.3 [69] using the lme4 package for mixed-effects logistic regressions [70]. Data files and analysis scripts for all analyses including supplemental analyses are available on the Open Science Framework at https://osf.io/9bxpk/. Furthermore, all research procedures complied with APA ethical standards and were approved by the Institutional Review Board at the University of Chicago, where the data were collected. All participants provided informed consent to participate in this research and agreed to their data being used for analysis following debriefing. All consent and agreements with research participants were obtained online using IRB-approved electronic consent and debriefing forms.

### Experiment 1

#### Participants

U.S.-based participants (*n* = 502) were recruited online using MTurk. These participants were required to have an 95% HIT approval rate and a minimum of 100 completed HITs. No other demographic or performance-based inclusion criteria were used for this study. MTurk samples tend to generate responses that are similar in reliability relative to data from lab-based samples [71–73]. Additionally, MTurk provides a diverse participant pool in terms of age, ethnicity, and SES, something that is not always the case in American university participant pools [74]. For Experiments 1 and 2, participants received a payment of $2.00 USD after completing all parts of the experiment. Prior to analysis, we excluded data from three participants who either declined to authorize the use of their data for analysis or who failed to respond to the post-debrief data authorization question. Additionally, all trials with response latencies under 150 ms, resulting in the removal of 34 trials. Five participants with four or more of these latency-based exclusions were removed entirely from analysis, resulting in the removal of an additional 16 trials. These latency-based exclusions were implemented in both experiments to avoid the inclusion of physiologically implausible responses, responses made in the absence of deliberation, and the participants who made such responses for at least half of their trials. No other exclusion criteria were implemented. After these exclusions, the final sample consisted of 494 participants (265 Female, 228 Male, 1 Non-Binary; *M*_*age =*_ 35.0 years; *SD*_*age*_ = 10.2 years; *Range*_*age*_ = 18–70 years).

Our sample size was based on previous work by FeldmanHall and colleagues [21]. Our final sample exceeded the largest participant group reported in their experiments. Using PANGEA (jakewestfall.org/pangea/), we estimated that our 2 (SES: low, high) × 2 (Inequity: low, high) within-participants design (with one trial per condition) and final sample were adequately powered to detect an effect as small as *d* = 0.164, 1–*β* = .804, assuming a default variance parameter value, *var*(error) + *var*(participants*SES*inequity) = 0.417.

#### Stimuli

Eight White male faces from the Chicago Face Database [75] were chosen as stimuli for the modified Ultimatum Game described below (i.e., Justice Game). The eight faces were divided into two groups of four faces (i.e., one group per SES level) and equated for trustworthiness, attractiveness, and age based on pre-existing ratings from the Chicago Face Database. Trustworthiness and attractiveness ratings from the Chicago Face Database were provided on a 7-point scale from 1 (Not at all) to 7 (Extremely). Age ratings were provided as estimates (in years). The resulting two groups each consisted of four male faces that were somewhat below average in trustworthiness (*M*_*1*_ = 3.17, *M*_*2*_ = 3.25) and attractiveness (*M*_*1*_ = 3.24, *M*_*2*_ = 2.86), all two-tailed independent-samples |*t*(6)|<0.650, *p*>.54. Faces from both groups were perceived as being approximately 27 years old (*M*_*1*_ = 27.0, *M*_*2*_ = 27.2), two-tailed independent-samples *t*(6) = -0.065, *p* = .95. Faces belonging to the same group were superimposed on the same color background (i.e., blue or red) to indicate that face’s SES level (see below). SES–color associations were counterbalanced across participants.

Previous research has relied on various antecedents of status including clothing, ascribed occupation/income/rank, body posture, facial structure, and even car ownership [39]. Although such cues may afford ecological validity in some contexts, these status cues do not always unambiguously convey status level. Moreover, perceptual antecedents of status such as clothing are demonstrably confounded with important social dimensions like competence [76,77], making it difficult to reliably isolate effects of status. To avoid these potential pitfalls in our initial exploration of how status shapes justice decision making, we therefore used our existing procedure for ascribing status levels through learned color-coded background cues [78–82].

#### Procedure

Upon consenting to participate, participants first completed the SES–color association learning task (see below). Participants then read through some brief instructions describing the Justice Game and their role as Player C. Throughout the instructions, participants completed comprehension items that required the correct response in order to proceed (see S1 Text). To begin the main block of trials, participants were instructed to place their hands on the S, D, and F keys. After completing the Justice Game, participants completed several exploratory measures and demographic questions (see S4 Text). After completing these measures, participants were debriefed and given a completion code to receive compensation for completing the experiment.

#### Learning status-color associations

Prior to completing the Justice Game, participants first learned to associate the colors blue and red with different levels of SES (i.e., high or low). Both the SES–color association training and the subsequent Justice Game were presented online via Inquisit 4 Web (Version 4.0.9: Millisecond Software, Seattle, Washington). Participants initially read the following definitions of SES: “Those who have the HIGHEST social status tend to have the most money, the most education, and the most respected jobs. Those who have the LOWEST social status tend to have the least money, the least education, and the least respected jobs or no job.” Following these definitions, participants learned that they would see pictures of low- and high-SES individuals in the U.S., and that the pictures would be superimposed on a colored background denoting their SES level (e.g., blue = low SES, red = high SES). SES–color associations were counterbalanced across participants.

To thoroughly learn SES–color associations, participants completed simple association training blocks. In an initial block of 12 trials, participants passively viewed images of darkened silhouettes over a colored background (i.e., red or blue) paired with a sentence describing the silhouette’s color-specific SES level (6 trials per SES level). Next, participants completed a block of 36 trials in which they viewed the same color-framed silhouettes without ascribed SES information (other than the colored background) and responded to a prompt regarding the silhouette’s SES level (e.g., “Does this color mean HIGH or LOW status in the US?”). Participants had unlimited time to press 1 for high SES or 2 for low SES. Incorrect responses elicited an error message: “INCORRECT—please give the correct response in order to proceed”. At the end of the block, participants received feedback on their overall accuracy and instructions that they would repeat the preceding block, irrespective of their initial accuracy. Any errors resulted in repetition of this training block. Training concluded with the next successful completion of the training block at 100% accuracy. On average, participants needed 1.3 blocks to reach 100% accuracy. Training data from two participants was unavailable. Although the present study did not test recall of status–color associations at the end of the experiment, previous research using this training procedure with relatively long cognitive tasks has shown good retention (~89%) and few differences when analyses were conducted with or without participants failing the post-task manipulation check [78].

#### Third-party modified ultimatum game

Based on the paradigm from FeldmanHall and colleagues [21], participants played the Justice Game as a third party with the power to redistribute resource allocations between two other individuals. On each of eight trials, participants decided whether and how to redistribute monetary splits that were proposed by an independent allocator (i.e., Player A). For each split, Player A divided $1 USD between himself and a recipient (i.e., Player B). Participants were led to believe that Players A and B were real people and that Players A (i.e., a new Player A for every trial) made their allocations as part of a previous experiment (see S1 Text for full task instructions). Moreover, each participant was told that Players A and B would receive bonus payouts via MTurk as a direct result of the participant’s decisions. In reality, allocator decisions were fixed. Player A’s offers were always inequitable but varied in the degree to which they advantaged Player A versus Player B. Possible selfish offers were $0.80/$0.20 and $0.60/$0.40. Possible hyper-generous offers were $0.20/$0.80 and $0.40/$0.60. Although not the main focus of the present paper, we included hyper-generous offers (i.e., those favoring the recipient more than the allocator) because these offers allow the opportunity to examine inequity in the absence of a clear victim (see S3 Text for analyses).

After viewing each monetary split, the participant (i.e., Player C) was then asked to determine the final monetary outcomes of Players A and B, choosing one of three options: accept, compensate, or reverse. The accept option maintains the status quo of Player A’s original offer. In this case, the participant (i.e., Player C) accepts the monetary split between Players A and B that was offered by Player A. The compensate and reverse options signify different redistribution schemes. The compensate option bonuses whichever player received the least amount of money, increasing that player’s earnings to match the amount received by the player initially advantaged by the allocator’s (i.e., Player A) offer, thereby raising and equalizing the offer amounts between Players A and B. The reverse option flips the monetary split made by the allocator (i.e., Player A), effectively reversing the fortunes of Players A and B. Critically, the implications of the reverse option depend on the relative selfishness or generosity of the allocator’s (Player A) offer. If the allocator (i.e., Player A) makes a selfish offer (e.g., $0.80/$0.20), then a reverse decision would punish Player A and reward the recipient of the selfish offer (i.e., Player B). Because our primary focus is on these selfish offers, we refer to the reverse option as the “punish” decision in the results section. If Player A makes a hyper-generous offer (e.g., $0.20/$0.80), then a reverse decision would reward Player A but at the expense of Player B who was initially advantaged by the generous offer. For exploratory analyses and extended discussion of hyper-generous offers, see S3 Text.

**Fig 1:**
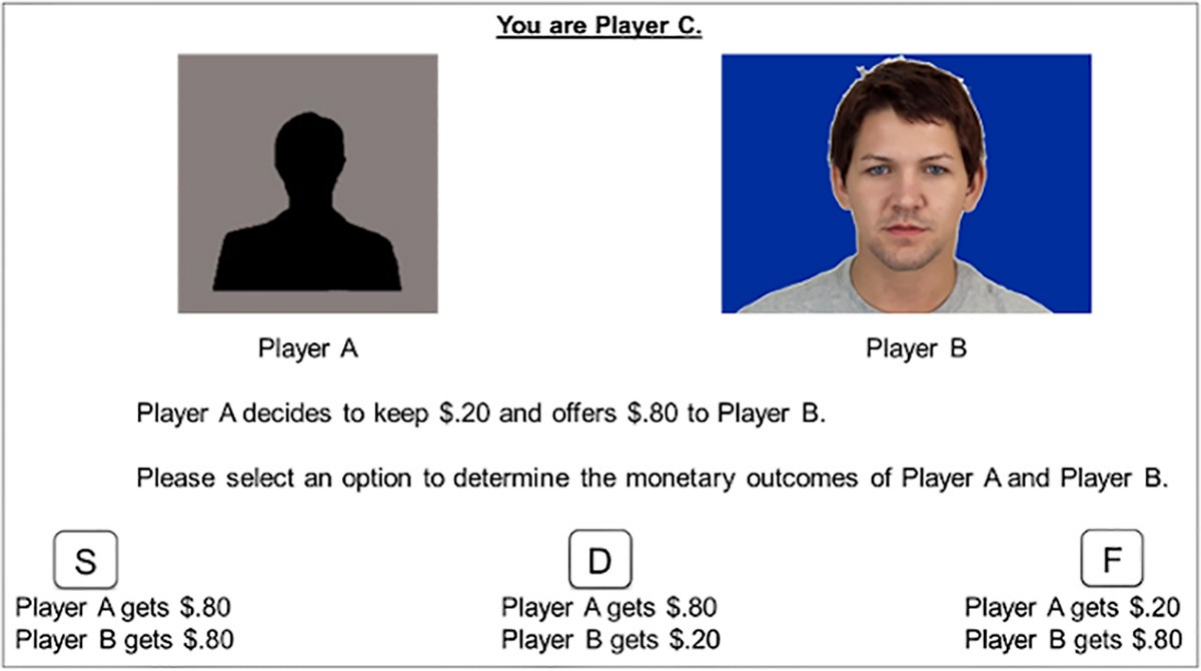
Example trial from the justice game from Experiment 1. Here, the allocator (Player A) makes an extremely generous offer to the recipient (Player B). For exploratory analyses and extended discussion of hyper-generous offers, see S3 Text. The SES associated with the colored background for Player B was learned as part of the SES–color association training prior to the Justice Game. The colors representing high and low SES were counterbalanced across participants.

Every trial began with a 250-ms fixation followed by the decision screen that remained until the participant responded. On the decision screen (see Fig 1), the allocator (i.e., Player A) was presented as a black silhouette of a face on a grey background. The recipient (i.e., Player B) was presented immediately to the right of Player A as a White male face framed by the color indicative of the face’s SES level. Above the two faces was a consistent reminder to the participant that she/he was Player C. Immediately below the two faces, participants learned of Player A’s initial allocation (e.g., “Player A decides to keep $0.80 and offers $0.20 to Player B.”) Beneath this sentence, participants reviewed their response options, represented by three different keys (e.g., S–accept, D–reverse, and F–compensate). In the instructions, participants learn that the meaning of each key varies from trial to trial. To help avoid confusion regarding the changing meanings of the response keys, participants viewed the concrete consequences of each response key (e.g., “Player A gets $0.80, Player B gets $0.20”) rather than its abstract representation (e.g., “accept”). The next trial began as soon as the participant made a response.

The trial sequence proceeded according to one of six randomly selected pseudorandom orderings intended to limit the repetition of factor levels (see S1 Text). Each sequence presented an equal number of low- and high-SES recipients as Player B and each of the four possible allocations per SES level for a total of eight trials. The Justice Game concluded after the eighth trial.

#### Data analysis

Because the psychological meaning of redistribution decisions differs depending on whether the allocator’s offer is selfish (i.e., allocating less to the recipient than to the allocator) or hyper-generous (i.e., allocating more to the recipient than to the allocator), we analyzed selfish and hyper-generous allocations separately. Analyses of decisions to punish selfish offers are reported in the main results section (see S2 Text for analyses involving all other decisions for selfish offers), and exploratory analyses of hyper-generous offers are reported in S3 Text.

In separate mixed-effect logistic regressions, we tested how decisions to punish (punish = 1) versus not punish (compensate = 0, accept = 0) were predicted by offer inequity (extremely selfish = 1, moderately selfish = -1), recipient SES (high SES = 1, low SES = -1), and the SES × Inequity interaction. All predictors varied within participants. The threshold for significance for Experiments 1–2 was set at *p* < .05. To the extent possible, we allowed for between-participants variance in intercepts and slopes for all within-participant factors (i.e., random effects) and the correlations between these random effects. However, sometimes, models failed to converge or were over-fitted [83]. When a model failed to converge or was over-fitted, we followed the steps outlined in S5 Text. If the original maximal model converged and was not over-fitted, then we adopted that model in line with existing recommendations [84]. The random effects structures for all reported models can be viewed in the study analysis script available on the Open Science Framework at https://osf.io/9bxpk/.

To investigate significant interactions, we conducted follow-up models on subsets of data corresponding to each level of each factor (Tables 1, 3 and 5) and each cell (Tables 2, 4 and 6) implicated in the interaction. As for the omnibus model, we attempted to fit as many random effects and correlation parameters as possible, following the aforementioned procedures. Because these follow-up models were restricted to decomposing significant interactions, we did not include any corrections for multiple comparisons.

**Table 1:** Simple effects for punitive versus non-punitive decisions in Experiment 1.

Contrast	Subset	*b*	*SE*	*CI*_*95%*_	*z*	*p*	
High vs. Low Inequity	High SES	0.785	0.115	[0.560, 1.011]	6.829	**<**	**.001**
	Low SES	0.878	0.113	[0.656, 1.099]	7.752	**<**	**.001**
High vs. Low SES	High Inequity	-0.441	0.104	[-0.644, -0.237]	-4.245	**<**	**.001**
	Low Inequity	-0.641	0.174	[-0.982, -0.300]	-3.686	**<**	**.001**

**Table 2:** Mean preference for punishment by condition in Experiment 1.

Offer Inequity	Recipient SES	*b*	*SE*	*CI*_*95%*_	*z*	*p*	
High	High	-0.579	0.094	[-0.764, -0.394]	-6.146	**<**	**.001**
	Low	-0.199	0.091	[-0.377, -0.022]	-2.203	** **	**.028**
Low	High	-1.529	0.119	[-1.763, -1.295]	-12.830	**<**	**.001**
	Low	-1.210	0.108	[-1.422, -0.998]	-11.200	**<**	**.001**

Negative betas reflect an overall preference for non-punitive solutions.

**Table 3:** Simple effects for punitive versus non-punitive decisions in Experiment 2.

Contrast	Subset	*b*	*SE*	*CI*_*95%*_	*z*	*p*	
High vs. Low Inequity	High SES	0.926	0.111	[0.709, 1.143]	8.378	**<**	**.001**
	Low SES	0.936	0.124	[0.694, 1.179]	7.561	**<**	**.001**
High vs. Low SES	High Inequity	0.603	0.094	[0.419, 0.787]	6.420	**<**	**.001**
	Low Inequity	1.874	0.304	[1.279, 2.469]	6.173	**<**	**.001**

**Table 4:** Mean preference for punishment by condition in Experiment 2.

Offer Inequity	Allocator SES	*b*	*SE*	*CI*_*95%*_	*z*	*p*	
High	High	0.245	0.090	[0.068, 0.421]	2.718	** **	**.007**
	Low	-0.437	0.092	[-0.617, -0.258]	-4.772	**<**	**.001**
Low	High	-0.787	0.097	[-0.977, -0.598]	-8.147	**<**	**.001**
	Low	-1.513	0.116	[-1.740, -1.285]	-13.020	**<**	**.001**

Negative betas reflect an overall preference for non-punitive solutions

**Table 5:** Simple effects between experiments 1 and 2 for punitive versus non-punitive options.

Experiment 1	Experiment 2	*b*	*SE*	*CI*_*95%*_	*z*	*p*	
High SES	High SES	0.700	0.108	[0.487, 0.912]	6.463	**<**	**.001**
Low SES	Low SES	-0.244	0.103	[-0.445, -0.043]	-2.374	** **	**.018**
Low SES	High SES	0.389	0.099	[0.196, 0.583]	3.943	**<**	**.001**
High SES	Low SES	0.073	0.104	[-0.130, 0.277]	0.705	** **	.481

**Table 6:** Mean preference for punishment across experiments.

Experiment	SES	*b*	*SE*	*CI*_*95%*_	*z*	*p*	
Experiment 1	High	-1.915	0.247	[-2.399, -1.431]	-7.760	**<**	**.001**
	Low	-1.279	0.182	[-1.636, -0.921]	-7.017	**<**	**.001**
Experiment 2	High	-0.487	0.138	[-0.757, -0.216]	-3.526	**<**	**.001**
	Low	-1.838	0.246	[-2.319, -1.357]	-7.487	**<**	**.001**

Negative betas reflect an overall preference for non-punitive solutions.

Additionally, we also observed a greater tendency to punish when financial offers involved a high-SES (vs. low-SES) party or when these offers were highly (vs. moderately) unfair, as indicated by significant main effects of SES, *b =* 0.170, *SE =* 0.051, *CI*_*95%*_ *=* [0.071, 0.270], *z =* 3.353, *p* = .001, and offer inequity, *b =* 1.016, *SE =* 0.072, *CI*_*95%*_ *=* [0.874, 1.158], *z =* 14.051, *p* < .001, respectively. All other effects were non-significant, (*p*>.52).

### Experiment 2

#### Participants

U.S.-based participants (*n* = 513) were recruited online using MTurk. Eligibility criteria for Experiment 2 were the same as in Experiment 1 with the exception that participants from Experiment 1 were not eligible to participate in Experiment 2. Prior to analysis, we excluded data from one participant who declined to authorize the use of their data for analysis. Additionally, we excluded all trials with response latencies under 150 ms, resulting in the removal of 58 trials. Seven participants with four or more of these latency-based exclusions were removed entirely from analysis, resulting in the removal of an additional 18 trials. No other exclusion criteria were implemented. After these exclusions, the final sample consisted of 505 participants (273 Female, 230 Male, 2 Non-Binary; *M*_*age =*_ 34.5 years; *SD*_*age*_ = 9.85 years; *Range*_*age*_ = 18–70 years).

Our sample size was based on previous work by FeldmanHall and colleagues [21]. As in Experiment 1, our final sample exceeded the largest participant group reported in their experiments. Using PANGEA (jakewestfall.org/pangea/), we estimated that our 2 (SES: low, high) × 2 (Inequity: low, high) within-participants design (with one trial per condition) and final sample were adequately powered to detect an effect as small as *d* = 0.162, 1–*β* = .803, assuming a default variance parameter value, *var*(error) + *var*(participants*SES*inequity) = 0.417.

#### Design and analysis

**Fig 2:**
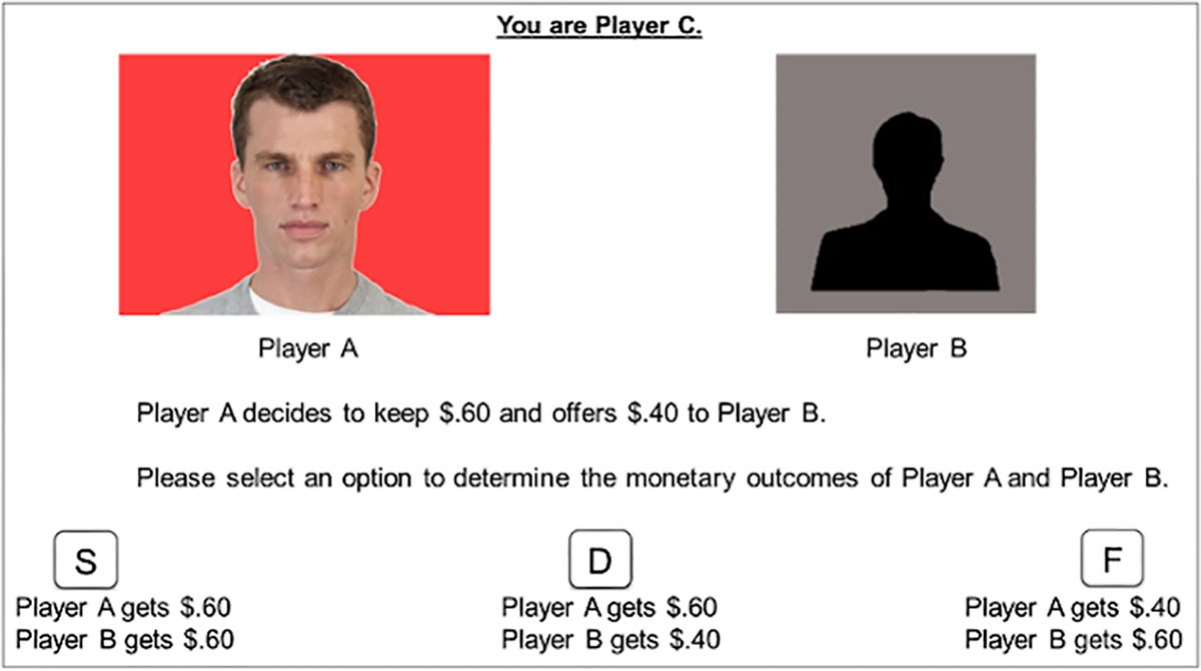
Example trial from the justice game from Experiment 2. Here, the allocator (Player A) makes a somewhat selfish offer to the recipient (Player B). The SES associated with the colored background for Player A was learned as part of the SES–color association training prior to the Justice Game. The colors representing high and low SES were counterbalanced across participants.

The face stimuli, status-color association training, Justice Game paradigm, and data analysis were the same as in Experiment 1. The only difference is that the recipient (i.e., Player B) was represented by a black silhouette and the allocator (i.e., Player A) was represented by the same face stimuli used in Experiment 1 (see Fig 2). In other words, Experiment 2 varied the SES level of the allocator rather than the recipient. Accordingly, the SES factor in all analyses for Experiment 2 refers to the SES level of the allocator rather than the recipient.

To facilitate comparisons across experiments, we combined the data from Experiments 1–2 and repeated the same omnibus model used for these experiments but with experiment as an additional between-participants predictor (experiment 2 = 1, experiment 1 = -1. The resulting model included predictors for all possible interactions between SES, offer inequity, and experiment. As for the preceding analyses, we allowed for between-participants variance in intercepts and slopes for all within-participant factors (i.e., random effects) and the correlations between these random effects. However, sometimes, models failed to converge or were over-fitted [83]. When a model failed to converge or was over-fitted, we followed the steps outlined in S5 Text. If the original maximal model converged and was not over-fitted, then we adopted that model in line with existing recommendations [84]. The random effects structures for all reported models can be viewed in the study analysis script available on the Open Science Framework at https://osf.io/9bxpk/.

## Results

In the following experiments, we differentiated between the competing accounts of how SES shapes decisions to address inequity in financial offers from Player A to Player B from the perspective of a third party (Player C) who has no stake in the exchange—the court of public opinion. Although participants generally preferred non-punitive compensation over punishment [21,22,65], we nonetheless observed sensitivity to SES and offer inequity when participants did opt to punish selfish allocators. Accordingly, our main analyses focused on how frequently participants chose to punish selfish offers as a function of the offer’s selfishness and the SES of the recipient (Experiment 1) or the allocator (Experiment 2). Using mixed-effects logistic regression, we explored how SES and inequity influenced decisions to punish.

### Experiment 1: Effects of recipient SES on third-party punishment

**Fig 3:**
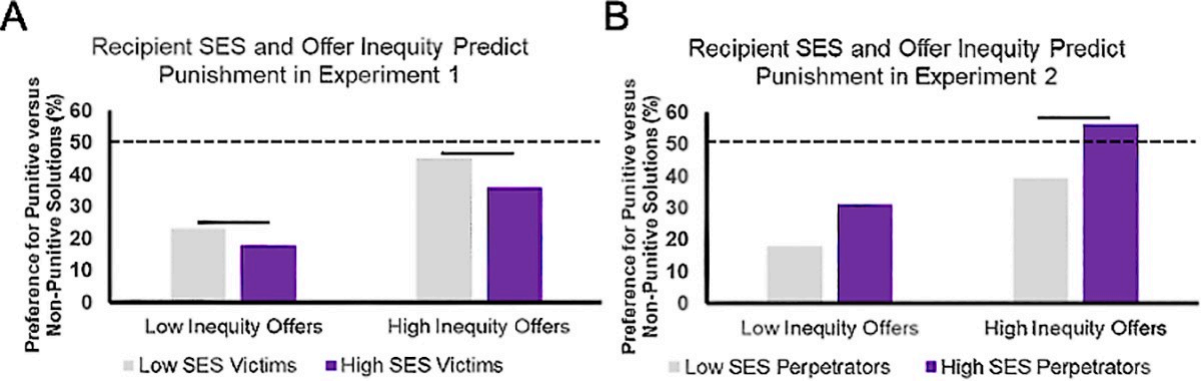
Percentage of decisions to punish by condition and experiment. The recipient’s and allocator’s SES levels were manipulated in separate experiments (panels A and B, respectively). For ease of interpretation, percentages from the raw count data are plotted rather than fitted log odds ratios. For this reason, error bars are not included in this figure. In both experiments, the percentage of decisions to punish (vs. not punish) perpetrators of selfish allocations is plotted as a function of ascribed SES (i.e., high, low) and offer inequity (i.e., highly selfish, less selfish). In Experiment 1 (A: *n* = 494), we observed greater punishment of perpetrators who allocated selfish offers to low-SES victims. This was especially pronounced when offers were highly selfish. In Experiment 2 (B: *n* = 505), we observed greater punishment of high-SES (vs. low-SES) perpetrators, but only when offers were highly selfish. Significant simple effects of SES are indicated with solid lines; all simple effects of offer inequity (i.e., contrasts of same-color bars) were significant (see Tables 1 and 3). The dotted line represents equal preference for punitive and non-punitive options—all conditions were significantly different from this line (see Tables 2 and 4).

In a first experiment, we manipulated the ascribed SES of the recipient of the unfair offers (i.e., Player B) while the SES of the allocator (i.e., Player A) was left unspecified. Results revealed an overall preference not to punish relative to non-punitive options, as indicated by a significant effect of the intercept, *b =* -22.975, *SE =* 0.750, *CI*_*95%*_ *=* [-24.445, -21.505], *z =* -30.633, *p* < .001. We also observed significant main effects of recipient SES, *b =* 3.196, *SE =* 0.588, *CI*_*95%*_ *=* [2.044, 4.347], *z =* 5.439, *p* < .001, and offer inequity, *b =* 9.328, *SE =* 0.392, *CI*_*95%*_ *=* [8.560, 10.096], *z =* 23.796, *p* < .001. However, both effects were implicated in a significant SES × Inequity interaction, *b =* -2.425, *SE =* 0.276, *CI*_*95%*_ *=* [-2.965, -1.884], *z =* -8.788, *p* < .001. Analyses of simple effects (see Table 1) indicated that the preference for punishment increased as a function of offer inequity for both low- and high-SES recipients, but this effect of inequity was especially pronounced (i.e., 22.4% larger) for low-SES recipients. In fact, not only did highly selfish offers made to low-SES recipients elicit the highest punishment rate, this also came the closest to eliminating the overall preference for non-punitive responses (see Fig 3A).

### Experiment 2: Effects of allocator SES on third-party punishment

In Experiment 1, we found that participants preferred to punish more if the allocator victimized a low-SES person than if the allocator victimized a high-SES person. Just as people seemed more concerned about Martin Shkreli’s victimization of the disadvantaged (those depending on affordable prescription drugs) than those of privilege (the hedge fund investors who trusted him with their money), this finding suggests that participants were more averse to the financial exploitation of an already disadvantaged individual than they were to the exploitation of a relatively privileged individual. Independent of the recipient’s SES, participants also preferred to punish more for increasingly selfish offers. These results, however, do not speak to the question of whether a similar SES-based bias exists for perpetrators of financial exploitation. Thus, in a second experiment, we manipulated the SES of the allocator (i.e., Player A). The SES of the recipient (i.e., Player B) was left unspecified.

Results revealed an overall preference not to punish versus punish, as indicated by a significant effect of the intercept, *b =* -26.276, *SE =* 0.950, *CI*_*95%*_ *=* [-28.138, -24.414], *z =* -27.659, *p* < .001. We also observed significant main effects of recipient SES, *b =* 7.979, *SE =* 0.389, *CI*_*95%*_ *=* [7.217, 8.741], *z =* 20.516, *p* < .001, and offer inequity, *b =* 8.187, *SE =* 0.388, *CI*_*95%*_ *=* [7.426, 8.948], *z =* 21.094, *p* < .001. However, both effects were implicated in a significant SES × Inequity interaction, *b =* -1.214, *SE =* 0.256, *CI*_*95%*_ *=* [-1.716, -0.711], *z =* -4.734, *p* < .001. Analyses of simple effects (see Table 2) indicated that the preference for punishment increased as a function of offer inequity for both low- and high-SES allocators, but this effect of inequity was especially pronounced (i.e., 17.5% larger) for high-SES allocators. In fact, not only did highly selfish offers made by high-SES allocators elicit the highest punishment rate, this was the only condition that significantly flipped the overall preference for non-punitive responses (see Fig 3B).

### Comparison of third-party punishment across experiments

To determine whether manipulating the recipient’s (vs. the allocator’s) SES differentially affected decisions to punish selfish offers, we formally compared results across the experiments. To facilitate this comparison, we included experiment and all possible interactions with experiment as predictors in our analyses. We conducted an additional mixed-effects logistic regression predicting how decisions to punish selfish offers by experiment were predicted by both SES and offer inequity (see Methods for details).

**Fig 4:**
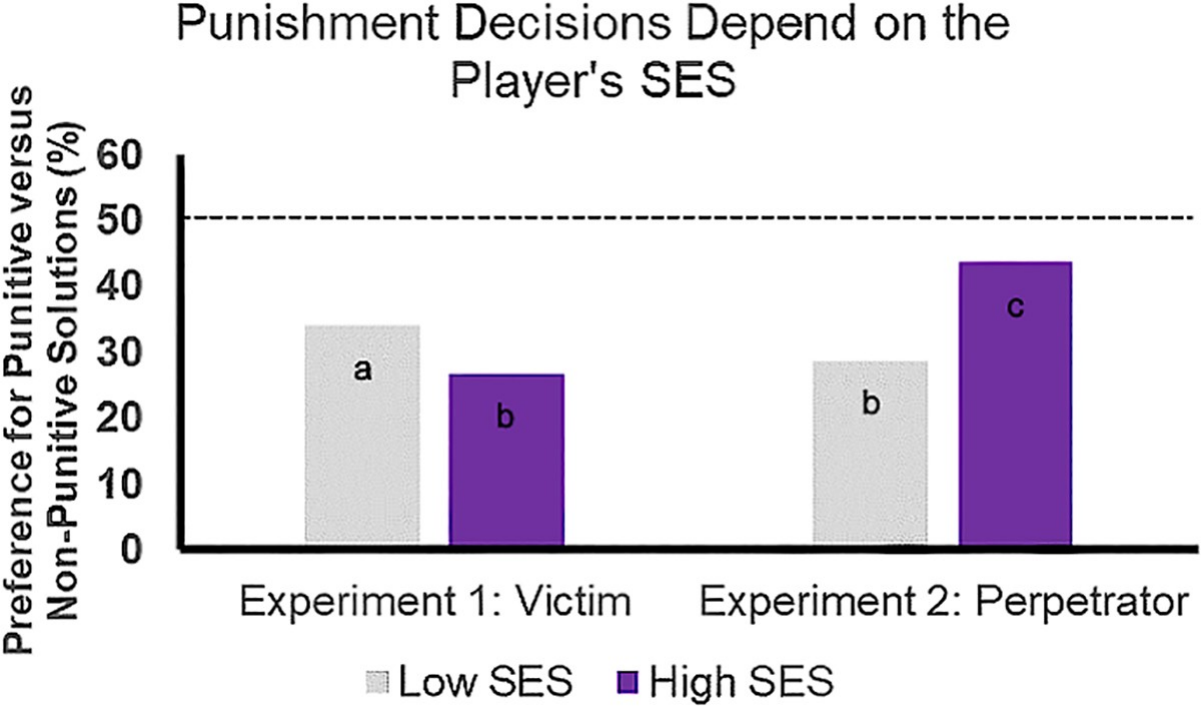
Formal comparison of decisions to punish between experiments. SES differentially shaped punishment decisions depending on whether SES varied for the recipient (Experiment 1) or the allocator (Experiment 2). For ease of interpretation, percentages from the raw count data are plotted rather than fitted log odds ratios. For this reason, error bars are not included in this figure. Independent of offer inequity, percentages of decisions to punish (vs. not punish) selfish offers are plotted as a function ascribed SES level (i.e., low, high) and experiment (i.e., Experiment 1, Experiment 2: total *n* = 999). Significant simple effects are indicated by distinct letters (i.e., c > a > b), all *p* < .05. See Table 5 for full contrast statistics. For all conditions plotted here, participants preferred non-punitive to punitive solutions, as indicated by all bars being significantly below the dotted line representing equal preference (see Table 6).

As in the preceding analyses, the results revealed a prevailing preference for non-punitive options, as indicated by a significant effect of the intercept, *b =* -1.565, *SE =* 0.125, *CI*_*95%*_ *=* [-1.810, -1.319], *z =* -12.496, *p* < .001. Results also indicated a greater overall tendency to punish in Experiment 2 than in Experiment 1, *b =* 0.283, *SE =* 0.103, *CI*_*95%*_ *=* [0.082, 0.485], *z =* 2.761, *p* = .006. This increase in punitive decisions in Experiment 2 was driven by exchanges involving a high-SES individual (see Table 3 for simple effects statistics), as indicated by a significant interaction between target SES and experiment on punishment decisions, *b =* 0.518, *SE =* 0.054, *CI*_*95%*_ *=* [0.413, 0.624], *z =* 9.625, *p* < .001. Tests of simple effects (Table 3) revealed that participants punished perpetrators with presumed higher standing relative to the victim more frequently. As depicted in Fig 4, we observed an increased preference to punish selfish allocators as a function of decreasing recipient SES (Experiment 1) and increasing allocator SES (Experiment 2).

## Discussion

The present findings show novel evidence in a controlled experimental context that social hierarchy can shape third-party perceptions of fairness even in a low-stakes financial allocation: Social hierarchy cues such as SES and the degree of inequity together influenced participants’ decisions to punish privileged allocators who made selfish offers. These results reveal that perceived social hierarchy information has important consequences for how inequity is mitigated. In line with past research [21–23,65,85], participants tended to more frequently punish allocators who made especially selfish offers. However, when participants did opt to punish selfish allocators, this was especially likely when the victim was low in SES or if the allocator was high in SES. This is consistent with our initial predictions that participants would be more likely to punish (a) those who exploit the already disadvantaged, and (b) privileged perpetrators of inequity. Moreover, this tendency to favor the low-SES party in punishment decisions was especially pronounced in the context of highly selfish offers (but see S2 Text) and for low-SES participants (see S6 Text). Nonetheless, preferential treatment of low-SES (vs. high-SES) individuals was observed even for participants reporting relatively high SES (see S6 Text), suggesting that effects cannot be entirely due to ingroup favoritism. Another central finding from this study is that participants more frequently opted for punishment when perpetrator’s SES was made explicit (viz., Experiment 2) rather than when the victim’s SES was made explicit (viz., Experiment 1). Only when a high-SES perpetrator made a highly unfair offer did a majority of participants prefer punishment over the non-punitive options, suggesting that these privileged individuals elicited more desire for punishment when they exploited others to fully maximize their share.

Taken together, the present findings illustrate that status cues add an additional level of inequity to already inequitable exchanges that can bias subsequent decisions to punish, and that social hierarchy serves as a meaningful backdrop against which decisions about fairness are made. These findings add to the literature on punishment of fairness violations in hierarchical contexts [13,46–51]. Previous work has so far focused largely on criminal offenders in an explicitly judicial context [e.g., 13,46,50,51], which may be biased by the salience of various stereotypes about criminality [52,53]. In general, these studies illustrate that participants desire to punish the rich more severely for exploitative criminal violations. Consistent with these findings, the present study also finds that high-SES perpetrators are punished more severely for their selfish offers. However, unlike these previous studies, this was found to be the case even in a context that minimized the salience of punishment—namely, in the absence of an explicitly judicial context or illegal behavior and when non-punitive means of restoring equity were available. In sum, the present findings rule out the possibility that preferences for punishing selfishness among the privileged are driven by ascribed illegal behavior in an explicitly judicial context where punitive outcomes are salient. Instead, punishing the privileged for selfishness may reflect social norms (e.g., *noblesse oblige*) that transcend contexts and cultures [15].

Other previous work suggests that encouraging people to think of the rich as having it all and/or the poor as miserable makes them more amenable to thinking about justice [42,67]. The present study goes beyond merely thinking about justice, ultimately providing evidence that salient examples of widening inequity between two individuals who are already unequal by virtue of their SES trigger punitive corrective action from third-party arbitrators. In Experiment 1, we observed an inclination to punish allocators who withheld money from an already economically disadvantaged low-SES victim. Moreover, participants were especially willing to punish a selfish allocator if that individual was ascribed a privileged, high-SES background (Experiment 2), which is consistent with work documenting our sensitivity to social rank [4–6,39] and egalitarian tendencies [1,2]. In other words, participants’ decisions to punish appear to reflect an aversion to concrete instances of widening inequality, both when the poor do not receive a fair share, but especially when the rich get richer at the expense of others. Although plausible, it remains to be seen whether this aversion is exacerbated when the status of both the perpetrator and victim is made salient.

These findings have important implications for judicial decision making in cases involving inequity in financial exchanges. Making the relative SES of plaintiffs and defendants explicit may bias jurors in favor of the party of lower SES. Although this appears to run counter to the principle that justice is blind to characteristics of parties involved in a given case, knowledge of the SES of plaintiffs/defendants may be particularly germane to decisions about the relative harm of financial inequity. Barring that information from the court may hinder the court’s ability to accurately assess the psychological and material harms of financial exploitation. For example, a monetary loss is likely to elicit greater distress and financial hardship in a low-SES individual than in a high-SES individual.

The present work helps to differentiate between two possible accounts of how SES may shape third-party fairness decisions. A greater willingness to allow exploitation of the rich to go unpunished (see Experiment 1) is inconsistent with predictions stemming from lay beliefs that the rich are relatively law-abiding [86] and less accustomed to suffering [87] in comparison to the poor. Such beliefs would presumably make the victimization of high-SES individuals potentially more salient and deserving of punishment [61,64]. However, we instead observed greater punishment on behalf of low-SES compared to high-SES victims in Experiment 1. Additionally, beliefs that the rich are relatively good and less accustomed to suffering have been linked to more lenient punishments for rich perpetrators [88,89], which is the opposite of what we observed in Experiment 2. Instead, participants’ preferences to punish selfish high-SES allocators and protect low-SES recipients by punishing those who exploit them are more consistent with (a) violation of the norm that the rich should be generous with or at least not exploit the poor [15], and (b) work showing that the rich effectively pursue their own self-interest whereas the poor are generally perceived as well-intentioned, albeit less effective [46,67,90]. These latter group-based beliefs are tied to distinct motivated emotions, with people experiencing envy of the privileged and pity for those who are deemed well-intentioned but disadvantaged [90,91]. Although the present study did not directly examine participant emotions, previous work has implicated a significant role of affect in justice-related decision making. Particularly for envied classes, individuals show greater evidence of pleasure when members of these classes experience misfortune [68]. It is therefore possible that participants enjoyed punishing high SES allocators. A similar mechanism may also underlie the tendency for participants to favor punishment as a means of correcting the exploitation of low-SES recipients. Related to this possibility, one fMRI study showed that punishment (vs. compensatory redistribution) was associated with greater activity in brain regions involved in reward processing [23]. Accordingly, future work may explore whether the SES of the allocator/recipient may accentuate third-party preferences for punishment vis-a-vis associated emotional and reward responses.

It will be important for future work to explore whether the present findings may hold when more is at stake (e.g., greater sums of money) and/or after having met the individuals involved in the Justice Game exchanges in real life. For example, with a few exceptions [63], sociological data often show that wealthy (vs. poor) victims elicit greater, not lesser, penalties for criminal offenders [61,64]. One exciting future direction at the rich but often-overlooked nexus of sociology and social psychology [92] would be to explore when and how structural factors (e.g., aspects of the legal/judicial system that privilege the wealthy) can be overcome by the individual-level aversion to widening inequality evinced by the present findings. In other words, how can the presently reported effects of SES on perceived justice in individual interactions scale up to the society level? Americans (among other nationalities) tend to underestimate the magnitude of inequality at the level of society [4,45,93]. This misperception combined with ideological mobility beliefs [5,12,94], perceived competition between racial/ethnic groups [41,94–96] and dissatisfaction with the government [97,98], can diminish concern about the negative impacts of growing inequality [99], resulting in weak support for redistributive policies [9,12,94,97,98]. One means of catalyzing efforts to reduce inequality may be to make the issue more concrete. In the aftermath of the 2009 stock market crash, the Occupy Wall Street movement emerged under the slogan, “We are the 99%”. On the one hand, this slogan encapsulated in a few words ongoing inequities in American society where the wealthy elite wield a disproportionate share of capital and political influence relative to the poor and middle class [100]. On the other hand, this slogan was relatively abstract, making it easy for critics to misconstrue [101]. One line of research suggests that focusing on individuals (e.g., CEOs) rather than groups of people (e.g., corporations) may make punishments of fairness violations more appealing [102]. Accordingly, one means of increasing support for redistributive policies (punitive or non-punitive) may be to frame inequality as an interaction between two people: one representing the “haves” (i.e., the allocators) and the other representing the “have nots” (i.e., the recipients) within the broader American social hierarchy. In sum, the present findings highlight the important role of social hierarchy in the perception of fairness, with critical implications for real-world retributive and restorative justice.

## Supporting information

S1 Text Justice Game design and instructions. (DOCX)

S2 Text Preferences for redistribution over acceptance of selfish offers. (DOCX)

S3 Text All analyses of hyper-generous offers. (DOCX)

S4 Text Exploratory measures and demographic items. (DOCX)

S5 Text Standard model optimization procedure. (DOCX)

S6 Text Exploratory analyses of subjective SES. (DOCX)
