# Supplementary material for: Punishing the privileged: Selfish offers from high-status allocators elicit greater punishment from third-party arbitrators
Source: PLoS One. 2020 May 14;15(5):e0232369. doi: 10.1371/journal.pone.0232369 (PMC7224526; doi:10.1371/journal.pone.0232369)
Supplement: S1 Text — (DOCX) [file pone.0232369.s001.docx]

# Supplemental Text S1: Justice Game Design and Instructions

## Task Instructions

In this section, we provide a step-by-step overview of the Justice Game task instructions with accompanying figures. A breakdown of the instructions by screen is provided in Figures S1–S4 and Figures 1–2 in the main text.


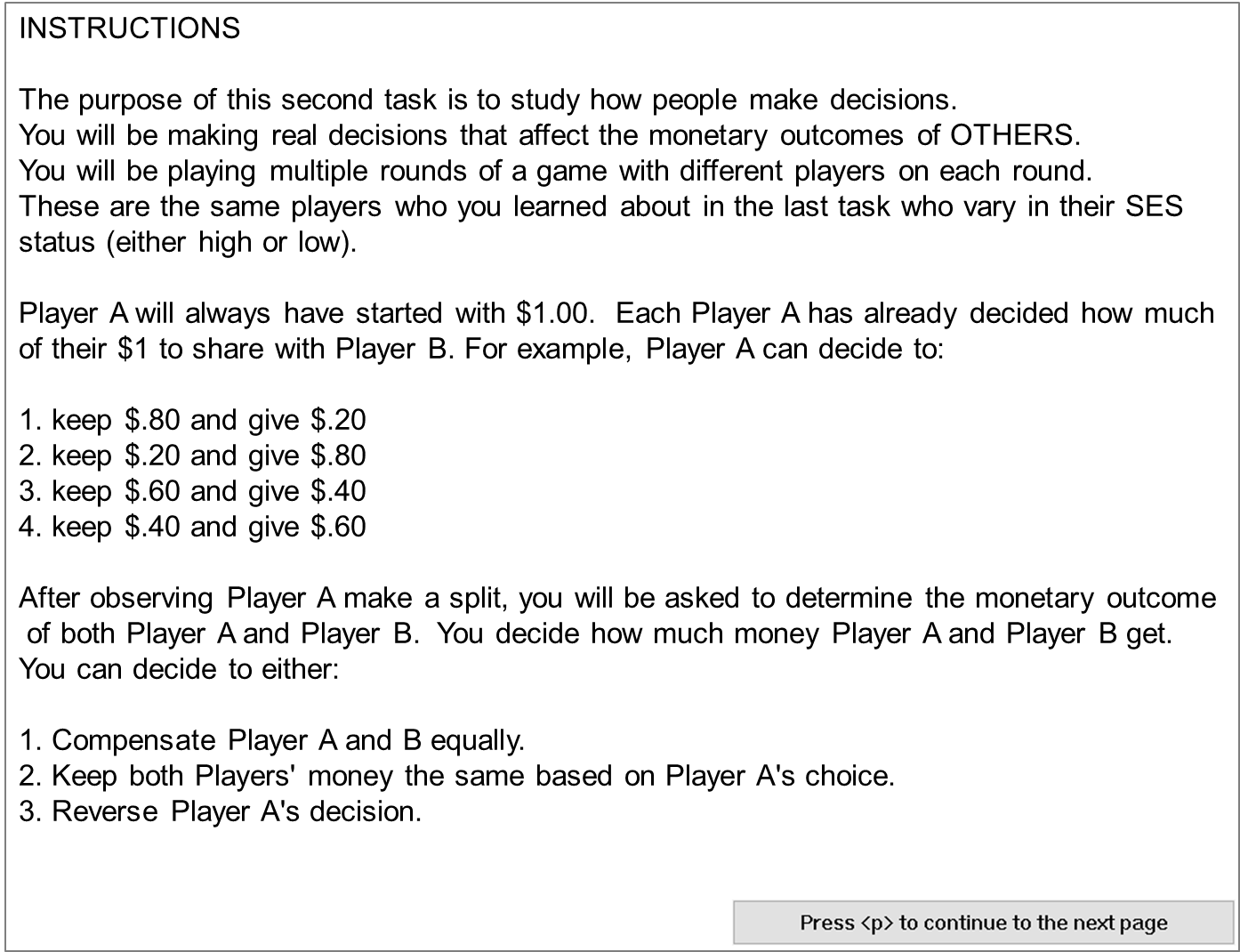


*Figure S1.* Initial instructions page following the status–color association training described in the main text. This page is the same for Experiments 1–2.


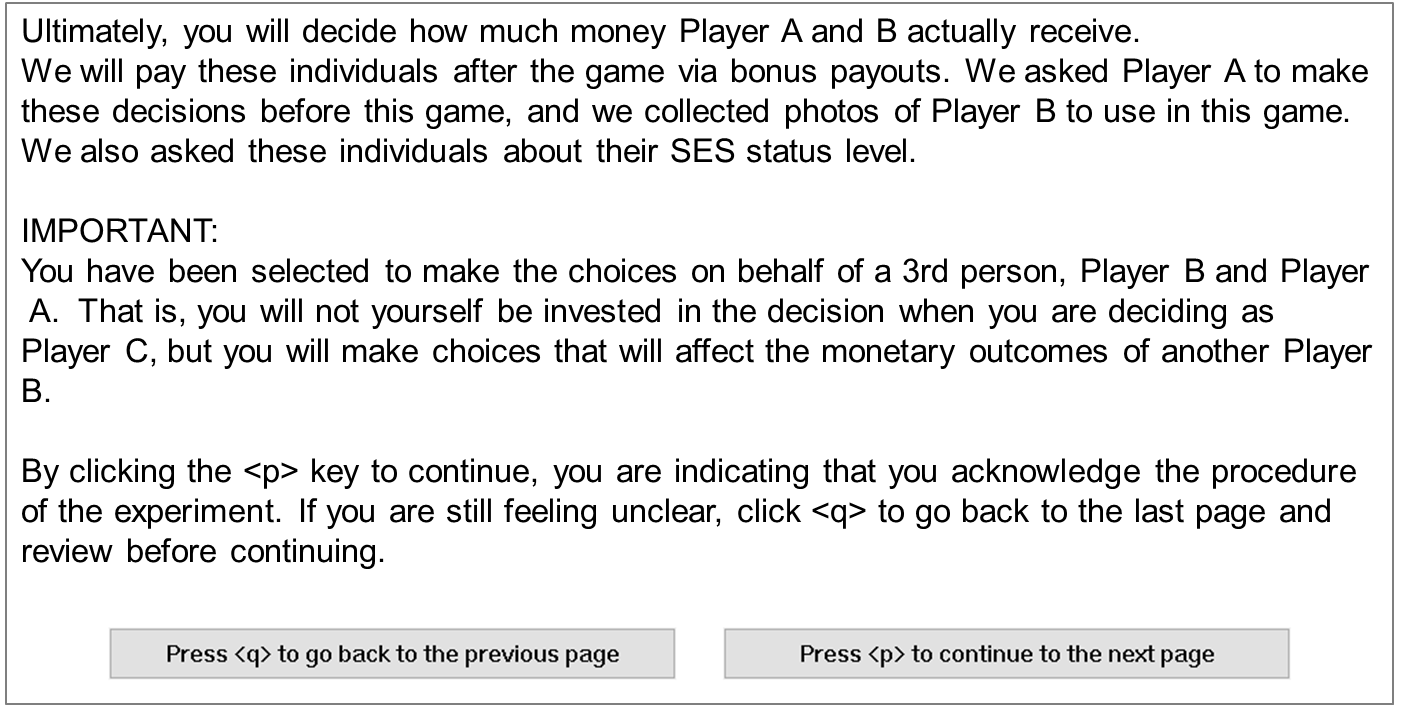


*Figure S2.* Second page of instructions from Experiment 1. Instructions are the same for Experiment 2 with the exception that photos were used for Player A, not Player B (see first paragraph).


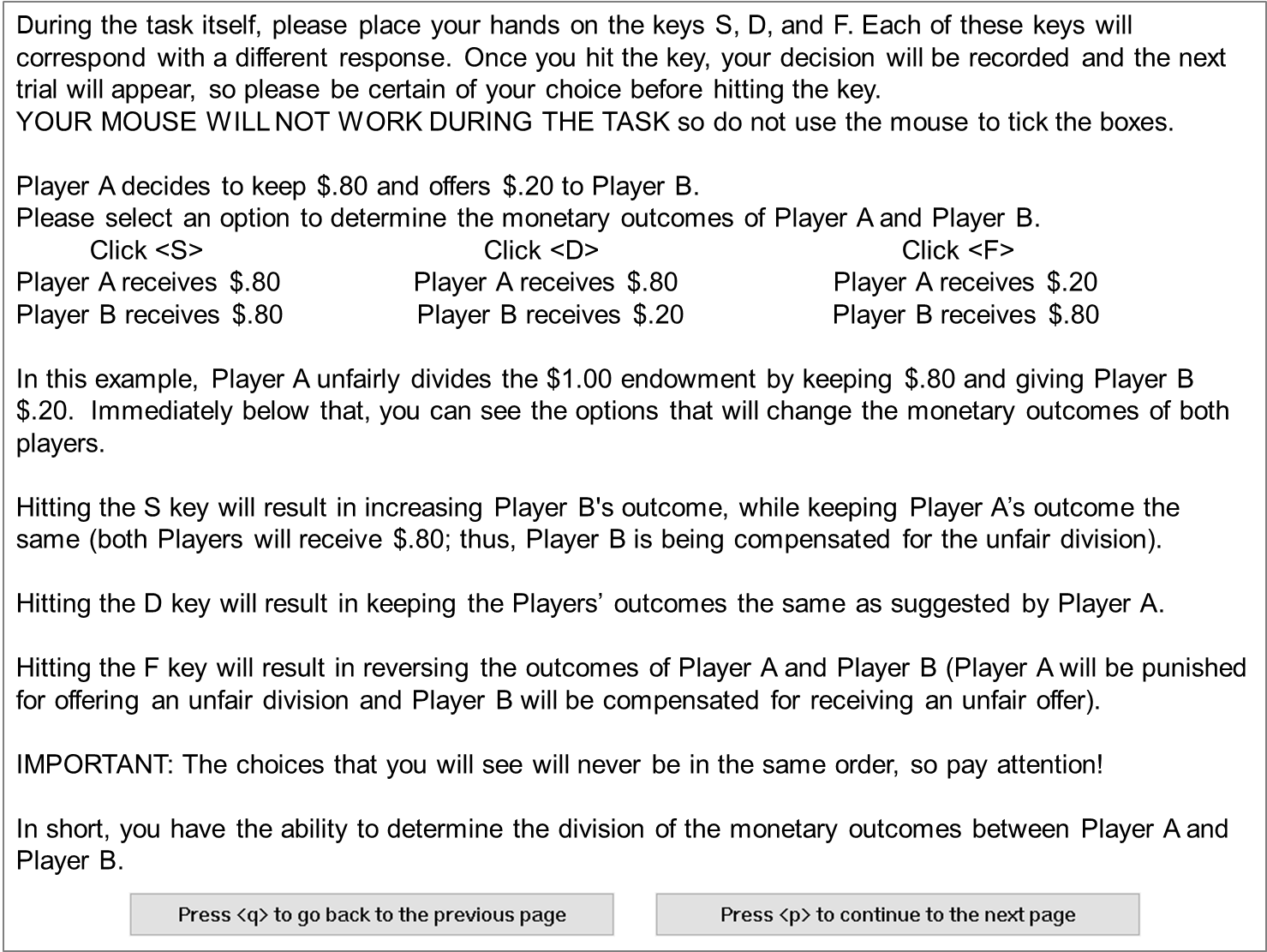


*Figure S3.* Third page of instructions, which provides an example of a possible offer from Player A and the participant’s options as Player C. This page was the same for Experiments 1–2.

After some initial instructions (Figures S1–S2) and an example of a trial (Figure S3), we checked participants’ comprehension with the following question: “According to the example in the instructions where Player A decides to keep $.80 and offer $.20 to Player B, how much money would Player B receive if you hit the S button?” Options were “$1.00”, “$.80”, and “$.20”. If participants were unsure, they were allowed to return to the preceding screen by pressing the “q” key. The instructions only proceeded once participants correctly answered this comprehension check question with the response option, “$.80”.

Next, participants were informed of the following: “At the end of the game, two trials from the task will be realized. That is, Players A and B will receive an additional BONUS for two of your decisions. This decision will be selected using a random number generator. If you selected option 1 in the previous example, Player B will be paid out an additional $.80 and Player A will be paid out $.80. If you selected option 2, Player B will be paid out an additional $.80 and Player A will receive an additional $.20, and so on. At the end, there will be a few short questionnaires to answer about your strategy. Please answer these carefully and truthfully. The session should take approximately 15 minutes. You will only be paid once you complete the session, and only if your answers appear valid. Note: THIS SURVEY IS FOR ACADEMIC PURPOSES SO PLEASE DO NOT TAKE THIS UNLESS YOU COMPLETE IT WITH MEANINGFUL ANSWERS.” Following this notice, participants responded to an additional set of comprehension checks (Figure S4). Upon completion of all comprehension checks, participants were notified that the economic game (i.e., the Justice Game) would begin on the following screen. Example trials from Experiments 1 and 2 are depicted in Figures 1 and 2 (see main text), respectively.


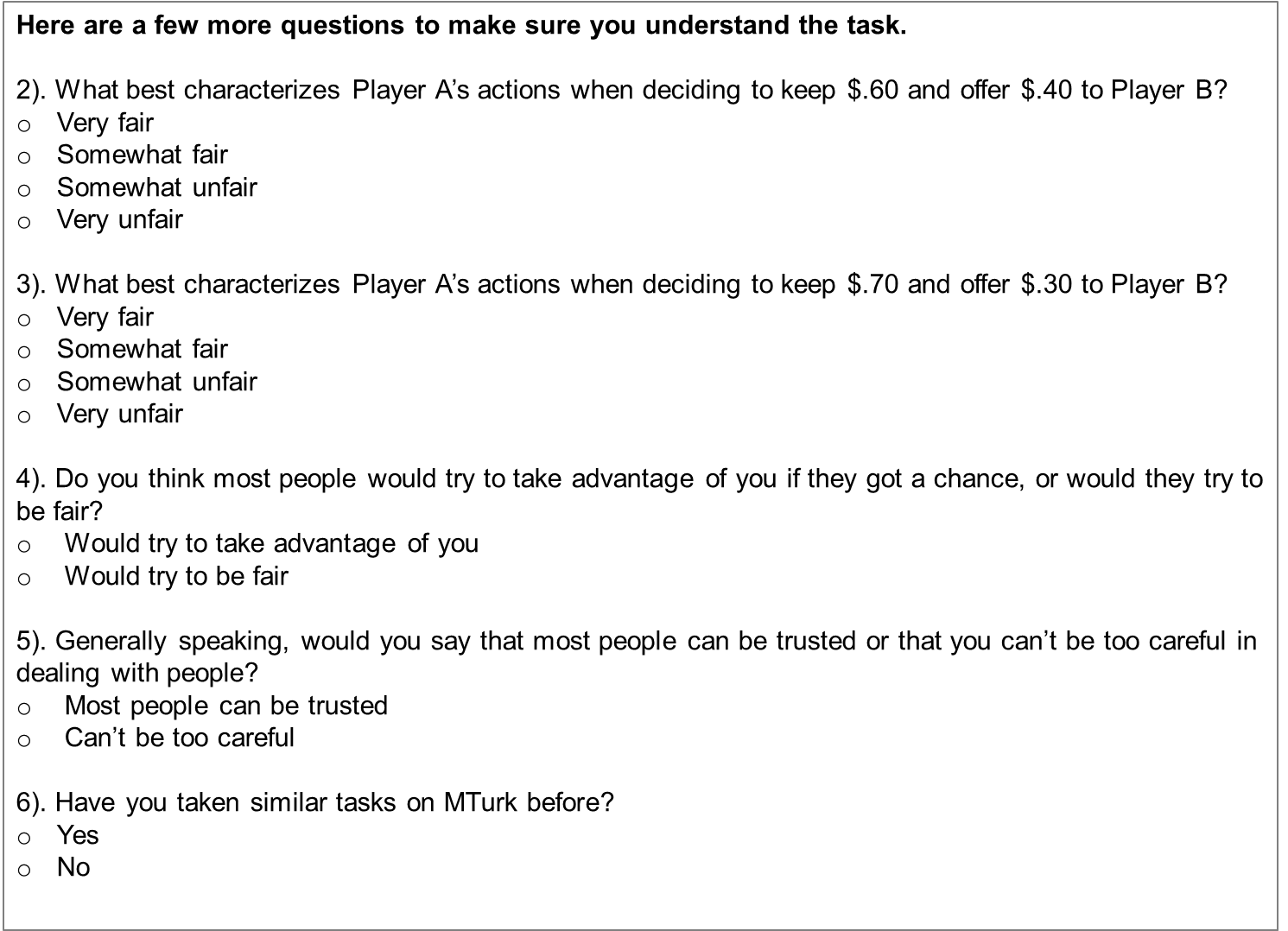


*Figure S4.* Additional comprehension check questions. The instructions would not proceed until the participant provided correct answers to question 2 (“somewhat unfair”) and question 3 (“very unfair”). Note that question 3 presents a hypothetical 70:30 split, but this split was never presented to any participant in the experimental block of trials.

## Pseudorandomized Trial Sequences

As reported in the main text, the eight-trial sequence proceeded according to one of six randomly selected pseudorandom orderings intended to limit the repetition of factor levels (see Table S1). Each sequence presented an equal number of low- and high-SES recipients as Player B^[[1]](#footnote-1)^ and each of the four possible allocations per SES level for a total of eight trials. The Justice Game concluded after the eighth trial.

1. This applies to Experiment 1 only. For Experiment 2, each sequence presented an equal number of low- and high-SES allocators as Player A and each of the four possible allocations per SES level for a total of eight trials. [↑](#footnote-ref-1)
