# Supplementary material for: Punishing the privileged: Selfish offers from high-status allocators elicit greater punishment from third-party arbitrators
Source: PLoS One. 2020 May 14;15(5):e0232369. doi: 10.1371/journal.pone.0232369 (PMC7224526; doi:10.1371/journal.pone.0232369)
Supplement: S2 Text — (DOCX) [file pone.0232369.s002.docx]

# Supplemental Text S2: Preferences for Redistribution over Acceptance of Selfish Offers

In this section, we report results from supplemental analyses of selfish offers for both experiments. For selfish offers, the allocator either acted quite selfishly, giving $0.20 and keeping $0.80, or only somewhat selfishly, giving $0.40 and keeping $0.60. Analyses reported here focus on participants’ preferences for redistribution over maintaining the status quo (i.e., accepting the allocator’s offer). Accordingly, we examined the preference to punish the allocator and reward the recipient by reversing the allocator’s offer (i.e., punish vs. accept) and the preference to merely compensate the recipient without punishing the allocator (i.e., compensate vs. accept). These analyses are followed by analyses examining how participants preferred to redistribute selfish offers, contrasting preferences for punishment and non-punitive compensation.

## Data Analysis for Selfish Offers

As a reminder, selfish offers were analyzed separately from hyper-generous offers (see Supplemental Text S3) because the psychological meaning of redistribution decisions differs depending on whether the allocator’s offer is selfish (i.e., allocating less to the recipient than to the allocator) or hyper-generous (i.e., allocating more to the recipient than to the allocator). For selfish offers, there were two levels of offer inequity, with extremely selfish offers coded as +1 and moderately selfish offers coded as -1. Recipient SES was similarly contrast coded: high=+1, low=-1. Each of three choice preferences were dummy coded (viz., punish vs. accept [punish=1, accept=0], compensate vs. accept [compensate=1, accept=0], and punish vs. compensate [punish=1, compensate=0]) and separately regressed onto recipient SES (low, high), offer inequity (low, high), and the SES × Inequity interaction. For each analysis (e.g., punish vs. compensate), the dataset included only responses relevant to that contrast, excluding non-focal responses (e.g., accept). To investigate significant interactions, we conducted follow-up models on subsets of data corresponding to each cell implicated in the interaction. Mixed-effects logistic regressions were implemented in R (R Core Team, 2019) using the lme4 package (Bates, Maechler, Bolker, & Walker, 2015).

## Experiment 1: Effects of Recipient SES on Redistribution Preferences

In a first experiment, we manipulated the SES of the recipient (i.e., Player B). The SES of the allocator (i.e., Player A) was left unspecified.

**Punish versus accept.** In this analysis, participants showed an overall preference to punish over accept for all selfish offers (see Figure S5), as indicated by a significant effect of the intercept, *b=*10.021, *SE=*0.952, *CI_95%_=*[8.154, 11.887], *z=*10.524, *p<*.001. Participants also showed a greater tendency to choose punish over accept with decreasing recipient SES (low SES: 69.6%; high SES: 55.5%) and increasing offer inequity (low inequity: 46.9%; high inequity: 75.4%), as indicated by main effects of recipient SES, *b=*-9.108, *SE=*0.995, *CI_95%_=*[-11.059, -7.158], *z=*-9.153, *p<*.001, and offer inequity, *b=*9.526, *SE=*0.995, *CI_95%_=*[7.576, 11.475], *z=*9.576, *p<*.001, respectively. Analyses of simple effects (see Table S2 for pairwise comparisons) revealed the overall preference to punish over accept was only reliable for high inequity offers (irrespective of recipient SES) and for low-SES recipients (irrespective of offer inequity). The SES × Inequity interaction was non-significant, *b=*0.136, *SE=*0.669, *CI_95%_=*[-1.175, 1.446], *z=*0.203, *p=*.839.

*
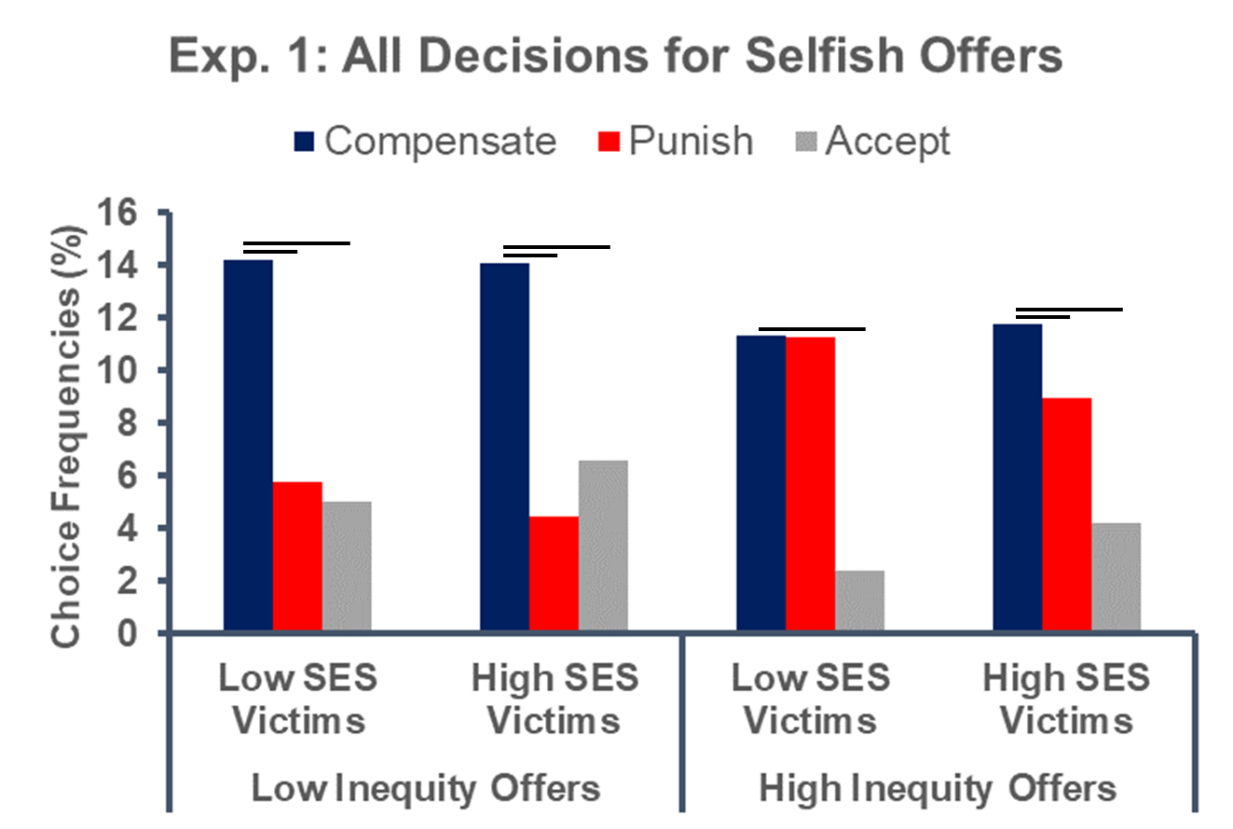
*

*Figure S5.* Percentage of choice frequencies (compensate, punish, or accept) for selfish offers are plotted as a function of offer inequity (i.e., low, high) and recipient SES (i.e., low, high). Analyses were conducted on count data, but percentages are plotted for ease of interpretation. For all possible decision contrasts with the compensate choice, we observed interactions between offer inequity and recipient SES. Significant simple differences between decisions are indicated with horizontal bars. See Tables S2–S4 for contrast statistics from Experiment 1.

**Compensate versus accept.** In this analysis, participants showed an overall preference to compensate over keep (see Figure S5), as indicated by a significant effect of the intercept, *b=*20.832, *SE=*0.914, *CI_95%_=*[19.040, 22.623], *z=*22.793, *p<*.001. We also observed significant main effects of recipient SES, *b=*-3.470, *SE=*0.608, *CI_95%_=*[-4.661, -2.279], *z=*-5.709, *p*<.001, and offer inequity, *b=*7.569, *SE=*0.646, *CI_95%_=*[6.303, 8.834], *z=*11.725, *p*<.001. However, both effects were significantly modulated by a SES × Inequity interaction, *b=*-2.543, *SE=*0.551, *CI_95%_=*[-3.622, -1.464], *z=*-4.619, *p<*.001. Analyses of simple effects (see Table S3 for pairwise comparisons) revealed that participants increasingly preferred to compensate over accept for highly selfish offers compared to moderately selfish offers. The magnitude of this effect was 63.2% larger when the recipient was low SES than when the recipient was high SES.

**Punish versus compensate.** Results revealed an overall preference to compensate over punish (see Figure S5), as indicated by a significant effect of the intercept, *b=*-22.513, *SE=*0.739, *CI_95%_=*[-23.962, -21.064], *z=-*30.452, *p<*.001. We also observed significant main effects of recipient SES, *b=*3.220, *SE=*0.803, *CI_95%_=*[1.647, 4.793], *z=*4.012, *p*<.001, and offer inequity, *b=*9.060, *SE=*0.396, *CI_95%_=*[8.284, 9.836], *z=*22.894, *p<*.001. However, both effects were significantly modulated by a SES × Inequity interaction, *b=*-2.382, *SE=*0.639, *CI_95%_=*[-3.635, -1.129], *z=*-3.726, *p<*.001. Analyses of simple effects (see Table S4 for pairwise comparisons) revealed that participants increasingly preferred to punish over compensate for highly selfish offers compared to moderately selfish offers. The magnitude of this effect was 10.5% larger when the recipient was low SES than when the recipient was high SES. Notably, the overall preference to compensate over accept was reliable for all combinations of recipient status and offer inequity, with the exception of low-status recipients of high-inequity offers. For these recipients, the preference for punishment compared to compensation was relatively similar (see Figure S5).

**Summary.** Preference for redistribution of either form (i.e., punish or compensate) relative to the status quo (i.e., accept) increased as a function of offer selfishness and decreased as a function of the recipient’s SES. The preference to compensate over accept for highly unfair offers was especially pronounced when the recipient was low in SES. Critically, when we directly compared the two forms of redistribution (i.e., punish vs. compensate), we found that participants still showed sensitivity to offer selfishness and the recipient’s SES in their relative preferences to punish allocators. Consistent with analyses of punish decisions reported in the main text, punishment preferences relative to non-punitive compensation were especially pronounced for low-SES victims of highly selfish offers.

## Experiment 2: Effects of Allocator SES on Redistribution Preferences

In Experiment 1, we found that participants generally preferred to resolve inequity through non-punitive compensation rather than punishment. However, as in the main text, participants’ punish decisions appeared to show less aversion to the financial exploitation of an already privileged high-SES individual than they were to the exploitation of an already disadvantaged low-SES individual. Although interesting, these results cannot speak to the question of whether a similar SES-based bias exists for perpetrators of financial exploitation. Thus, in a second experiment, we manipulated the SES of the allocator (i.e., Player A). The SES of the recipient (i.e., Player B) was left unspecified.

**Punish versus accept.** In this analysis, we observed an overall preference to punish over accept (see Figure S6), indicated by a significant effect of the intercept, *b*=4.924, *SE=*0.657, *CI_95%_=*[3.635, 6.212], *z=*7.489, *p<*.001. Participants showed a greater tendency to choose punish over accept with increasing allocator SES (low SES: 61.1%; high SES: 77.1%) and offer inequity (low inequity: 53.9%; high inequity: 82.6%), as indicated by main effects of allocator SES, *b=*1.118, *SE=*0.226, *CI_95%_=*[0.675, 1.561], *z=*4.947, *p<*.001, and offer inequity, *b=*4.552, *SE=*0.650, *CI_95%_=*[3.279, 5.825], *z=*7.006, *p<*.001, respectively. Notably, only for low-inequity selfish offers did we observe a relatively equal preference for punish relative to accept (see Table S5 for pairwise comparisons). The SES × Inequity interaction was non-significant, *b=*0.311, *SE=*0.227, *CI_95%_=*[-0.135, 0.757], *z=*1.367, *p*=.172.


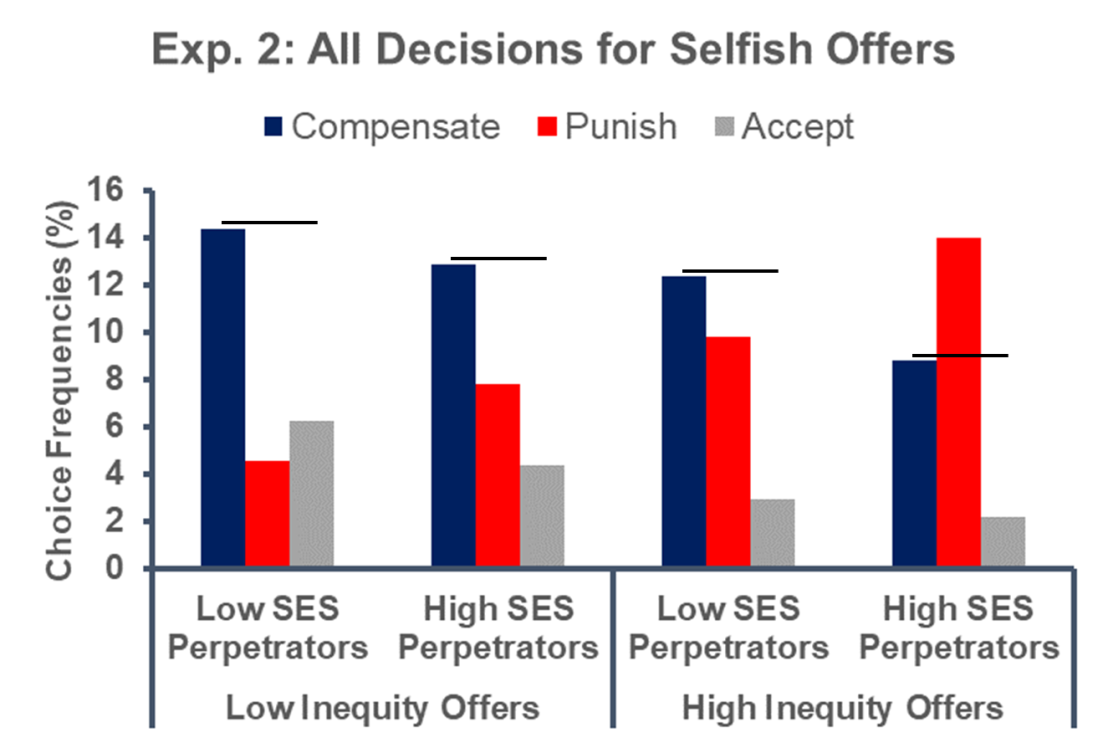


*Figure S6.* Percentage of choice frequencies (compensate, punish, or accept) for selfish offers are plotted as a function of offer inequity (i.e., high, low) and allocator SES (i.e., high, low). Analyses were conducted on count data, but percentages are plotted for ease of interpretation. Except for the compensate versus accept contrast, we observed independent main effects of offer inequity and recipient SES. For the compensate versus accept contrast, we observed an interaction between offer inequity and recipient SES. Significant simple differences between decisions are indicated with horizontal bars. See Tables S5–S7 for contrast statistics from Experiment 2.

**Compensate versus accept.** In this analysis, participants showed an overall preference to compensate over accept (see Figure S6), indicated by a significant effect of the intercept, *b*=27.780, *SE=*1.118, *CI_95%_=*[25.589, 29.971], *z=*24.846, *p<*.001. We also observed significant main effects of offer inequity, *b=*8.725, *SE=*0.697, *CI_95%_=*[7.359, 10.091], *z=*12.520, *p*<.001, and allocator SES, *b=*8.008, *SE=*0.714, *CI_95%_=*[6.608, 9.407], *z=*11.212, *p<*.001. However, both effects were modulated by a significant SES × Inequity interaction, *b=*1.748, *SE=*0.496, *CI_95%_=*[0.775, 2.720], *z=*3.523, *p<*.001. Simple effects analyses revealed that participants increasingly preferred to compensate over accept for highly selfish offers compared to moderately selfish offers. The magnitude of this effect was 99.4% larger when the allocator was low SES than when the allocator was high SES. See Table S6 for pairwise comparisons.

**Punish versus compensate.** Results revealed an overall preference to compensate over punish (see Figure S6), indicated by a significant effect of the intercept, *b=*-0.634, *SE=*0.144, *CI_95%_=*[-0.917, -0.350,], *z=-*4.386, *p<*.001. Participants showed a greater tendency to choose punish over compensate with increasing allocator SES (low SES: 35.0%; high SES: 50.2%) and offer inequity (low inequity: 31.2%; high inequity: 52.9%), as indicated by main effects of allocator SES, *b=*0.636, *SE=*0.082, *CI_95%_=*[0.476, 0.797], *z=*7.782, *p<*.001, and offer inequity, *b=*0.889, *SE=*0.097, *CI_95%_=*[0.699, 1.079], *z=*9.165, *p<*.001, respectively. Analyses of simple effects (see Table S7 for pairwise comparisons) revealed the overall preference to compensate over punish was eliminated for high-SES allocators (irrespective of offer inequity) and for highly selfish offers (irrespective of allocator SES). The SES × Inequity interaction was non-significant, *b=*0.022, *SE=*0.073, *CI_95%_=*[-0.122, 0.165], *z=*0.298, *p=*.766.

**Summary.** Replicating Experiment 1, results showed an overall preference for redistribution of selfish offers, either in the form of compensation or punishment. Preference for redistribution relative to the status quo (i.e., accept) increased as a function of offer selfishness and the allocator’s SES. However, participants were especially likely to compensate highly selfish offers if the offer was made by a low-SES (vs. high-SES) allocator. Consistent with findings reported in the main text, participants were more willing to punish the allocator if the allocator’s offer was highly selfish or if the allocator was high (vs. low) in SES. However, unlike in the main text, the effects of perpetrator SES and offer inequity were independent of one another. Critically, when we directly compared the two forms of redistribution (i.e., punish vs. compensate), participants still showed sensitivity to offer selfishness and the allocator’s SES in their relative preferences to punish allocators. As for the contrast with accept decisions, punishment preferences relative to non-punitive compensation increased as a function of offer selfishness and the allocator’s SES. Unlike in Experiment 1, where the general preference to compensate over punish was eliminated only for low-SES victim of a highly selfish offer, Experiment 2 revealed that this general preference was eliminated (i.e., both options were equally likely to be chosen) for highly selfish (vs. moderately selfish) offers and for high-SES (vs. low-SES) allocators.

## Comparison of Recipient and Allocator SES Effects

To determine whether manipulating the recipient’s (vs. the allocator’s) SES differentially affected preferences to punish or compensate allocations, we formally compared results across the experiments.

Results again revealed an overall preference to compensate over punish (see Figure S7), indicated by a significant effect of the intercept, *b=*-1.035, *SE=*0.134, *CI_95%_=*[-1.298, -0.773], *z=*-7.729, *p<*.001. For both recipient and allocator, we also observed an increase in punishment as an individual’s SES increased (low SES: 37.4%; high SES: 42.6%) and offer inequity increased (low inequity: 29.8%; high inequity: 49.9%), as indicated by significant main effects of SES, *b=*0.212, *SE=*0.059, *CI_95%_=*[0.096, 0.327], *z=*3.595, *p*<.001, and offer inequity, *b=*0.995, *SE=*0.087, *CI_95%_=*[0.824, 1.166], *z=*11.402, *p<*.001, respectively.

Critically, we observed two effects of the experiment-specific player for whom we varied SES (i.e., recipient in Experiment 1, allocator in Experiment 2). Participants showed a greater tendency to punish allocators (vs. non-punitive compensation) when the allocator’s SES was manipulated (i.e., Experiment 2: 42.7%) than when the recipient’s SES was manipulated (i.e., Experiment 1: 37.1%), as indicated by a significant main effect of experiment, *b=*0.291, *SE=*0.121, *CI_95%_=*[0.055, 0.528], *z*=2.414, *p*=.016. This increase in punitive decisions in Experiment 2 (vs. Experiment 1) was driven by exchanges involving a high-SES individual (see Table 3 for simple effects statistics), as indicated by a significant interaction between target SES and experiment on punishment decisions, *b=*0.509, *SE=*0.063, *CI_95%_=*[0.385, 0.632], *z=*8.071, *p*<.001. Tests of simple effects (Table S8) revealed that participants punished perpetrators with presumed higher standing relative to the victim more frequently. As depicted in Figure S7, we observed an increased preference to punish selfish allocators as a function of decreasing recipient SES (Experiment 1) and increasing allocator SES (Experiment 2). This increase in punitive decisions for high-SES allocators in Experiment 2 eliminated the overall preference for non-punitive compensation in other conditions. All other effects in this analysis were non-significant, all *p*>.56.


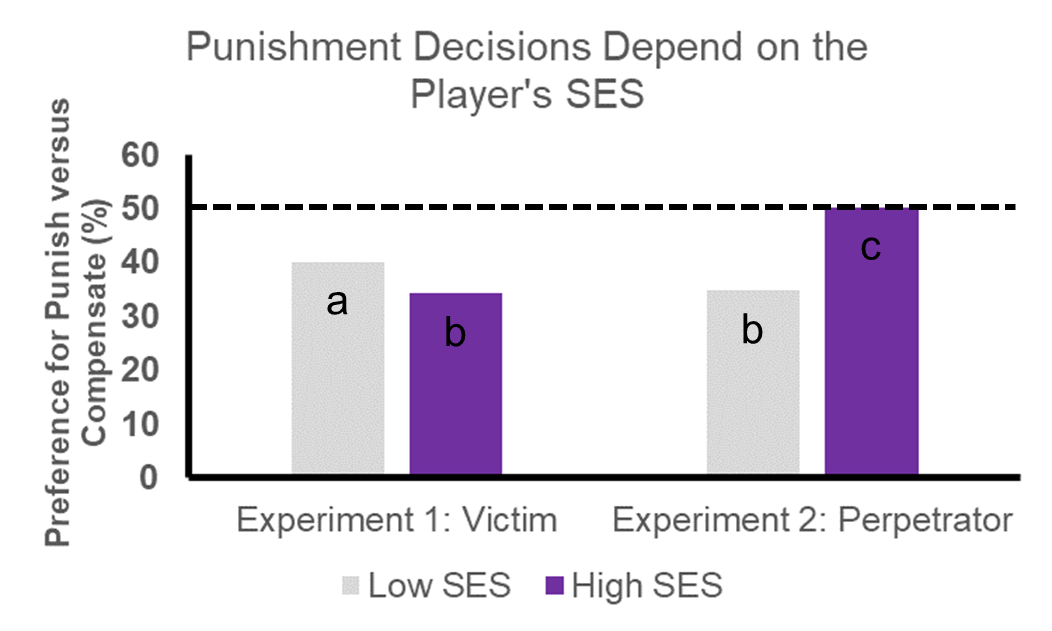


*Figure S7.* A formal comparison between experiments indicated that SES differentially shaped punishment decisions depending on whether SES varied for the recipient (Exp. 1) or the allocator (Exp. 2). Analyses were conducted on count data, but percentages are plotted for ease of interpretation. Independent of offer inequity, percentages of decisions to punish (vs. compensate) selfish offers are plotted as a function ascribed SES level (i.e., low, high) and experiment (i.e., Experiment 1, Experiment 2). Significant simple effects are indicated by distinct letters (i.e., c > a > b), all *p*<.05. See Table S8 for full contrast statistics. Except for the high-SES perpetrators in Experiment 2, participants preferred compensation to punishment, as indicated by these bars being significantly below the dotted line representing equal preference (see Table S9).

## Discussion

The finding that differences in an individual’s SES impacted how third-party arbiters chose to redistribute monetarily unfair and selfish offers reveals that perceived social hierarchy information has important consequences for how inequity is mitigated. In line with past research, participants generally preferred non-punitive over punitive redistribution options (i.e., compensate over punish, respectively); however, this preference was attenuated for highly inequitable offers (FeldmanHall, Sokol-Hessner, Van Bavel, & Phelps, 2014). We also observed marked increases in punishment decisions if the recipient (i.e., the victim) was low in SES or if the allocator was high in SES. These findings parallel those reported in the main text, but with one exception. In the main text, we found that punishment responses favored low-SES (vs. high-SES) victims/perpetrators and that this effect of status was enhanced when offers were highly inequitable. In these supplemental analyses, this interaction pattern between SES and offer inequity was only reliable for the contrast between punish and accept decisions in Experiment 1. Otherwise, effects of SES and offer inequity were independent of one another. Because the samples for these fine-grained analyses were smaller, it is not clear whether the discrepancies between the main and supplemental analyses are due to reduced power and/or a true difference between accepting the status quo and compensation. Further study is needed to delineate these possibilities. In conclusion, these supplemental findings in conjunction with those reported in the main text suggest that social hierarchy cues (i.e., SES) and the degree of inequity can both simultaneously influence participants’ preferences for redistribution.
