# Supplementary material for: Punishing the privileged: Selfish offers from high-status allocators elicit greater punishment from third-party arbitrators
Source: PLoS One. 2020 May 14;15(5):e0232369. doi: 10.1371/journal.pone.0232369 (PMC7224526; doi:10.1371/journal.pone.0232369)
Supplement: S3 Text — (DOCX) [file pone.0232369.s003.docx]

# Supplemental Text S3: All Analyses of Hyper-Generous Offers

In the context of inequitable distributions, hyper-generous offers in which the allocator keeps less than what is offered to the recipient are an important contrast to selfish offers in which the allocator keeps more than what is offered to the recipient. Although both selfish and hyper-generous offers involve an initially unequal distribution of resources, hyper-generous offers do not involve a clear victim. This is not the case for selfish offers where there is little ambiguity that the recipient (i.e., Player B) is a victim of the allocator’s (i.e., Player A) offer. In studies that focus solely on selfish offers, it is therefore not possible to disentangle effects of inequity from effects of victimization because these two constructs are confounded. To explore this distinction, the allocator’s offers in the present experiment were not always selfish. For half of the trials, participants (i.e., Player C) observed hyper-generous offers from the Player A in which Player B’s share was larger than that of Player A. As for selfish offers, hyper-generous offers varied in their extremity from moderate (Player A keeps $0.40 and Player B gets $0.60) to extreme (Player A keeps $0.20 and Player B gets $0.80).

We hypothesized that preferences for redistribution would differ qualitatively when inequitable offers were selfish versus generous. In contrast to our predictions for selfish offers (see main text), we anticipated that participants would mostly accept hyper-generous distributions or compensate the hyper-generous allocator. We anticipated that decisions to reverse the generous allocator’s decision (analogous to the punish decision for selfish offers) would be exceedingly rare because this undermines the allocator’s putative generosity, rewarding the allocator at the expense of the recipient. As for selfish offers, we anticipated that the perceived SES of Player A or B would heighten the salience of need or potential for generosity, making decisions to undermine the generous allocator especially rare for low-SES recipients (Experiment 1) and high-SES allocators (Experiment 2), respectively. This was motivated by previous work showing that very generous offers are more frequently rewarded (i.e., with compensation to the generous allocator) if the recipient is low SES or if the allocator is high SES (Bekkers & Wiepking, 2011; Hackel & Zaki, 2018).

For Experiments 1–2, we first report on participants’ preferences for redistribution over the status quo. Accordingly, we examined the preference to undermine allocator’s generous intent at the expense of the recipient (i.e., undermine vs. accept) and the preference to compensate the allocator at no expense to the recipient (i.e., compensate vs. accept). We then compared the relative preference for undermining the generous allocator (thereby harming the recipient of generosity) versus compensating the generous allocator without negatively impacting the recipient in redistribution decisions (i.e., undermine vs. compensate).

## Data Analysis for Hyper-Generous Offers

As a reminder, hyper-generous offers were analyzed separately from selfish offers (see Supplemental Text S2) because the psychological meaning of redistribution decisions differs depending on whether the allocator’s offer is selfish (i.e., allocating less to the recipient than to the allocator) or hyper-generous (i.e., allocating more to the recipient than to the allocator). For hyper-generous offers, there were two levels of offer inequity, with extremely generous offers coded as +1 and moderately generous offers coded as -1. Recipient SES was similarly contrast coded: high=1, low=-1. Each of three choice preferences were dummy coded (viz., undermine vs. accept [undermine=1, accept=0], compensate vs. accept [compensate=1, accept=0], and undermine vs. compensate [undermine=1, compensate=0]) and separately regressed onto recipient SES (low, high), offer inequity (low, high), and the SES × Inequity interaction. For each analysis (e.g., undermine vs. compensate), the dataset included only responses relevant to that contrast, excluding non-focal responses (e.g., accept). To investigate significant interactions, we conducted follow-up models on subsets of data corresponding to each cell implicated in the interaction. Mixed-effects logistic regressions were implemented in R using the lme4 package (Bates, Maechler, et al., 2015).

## Experiment 1: Effects of Recipient SES on Redistribution Preferences

In Experiment 1, we manipulated the SES of the recipient (i.e., Player A), leaving the SES of the allocator (i.e., Player B) unspecified.

**Undermine versus accept.** In this analysis, we observed an overall preference for accept over undermine (see Figure S8), as indicated by a significant effect of the intercept, *b=*-0.776, *SE=*0.214, *CI_95%_=*[-1.196, -0.356], *z=*-3.623, *p<*.001. However, participants showed a greater tendency to undermine over accept with increasing recipient SES (low SES: 32.9%; high SES: 44.4%) and offer inequity (low inequity: 33.8%; high inequity: 43.5%), as indicated by significant main effects of recipient SES, *b=*0.406, *SE=*0.130, *CI_95%_=*[0.152, 0.661], *z=*3.130, *p=*.002, and offer inequity, *b=*0.333, *SE=*0.130, *CI_95%_=*[0.078, 0.588], *z=*2.563, *p=*.010, respectively. These increases in preference to undermine over accept eliminated the overall preference for accept for high-SES recipients and high-inequity offers (see Table S10 for pairwise comparisons). The SES × Inequity interaction was non-significant, *b=-*0.088, *SE=*0.126, *CI_95%_=*[-0.335, 0.159], *z=*-0.698, *p*=.485.


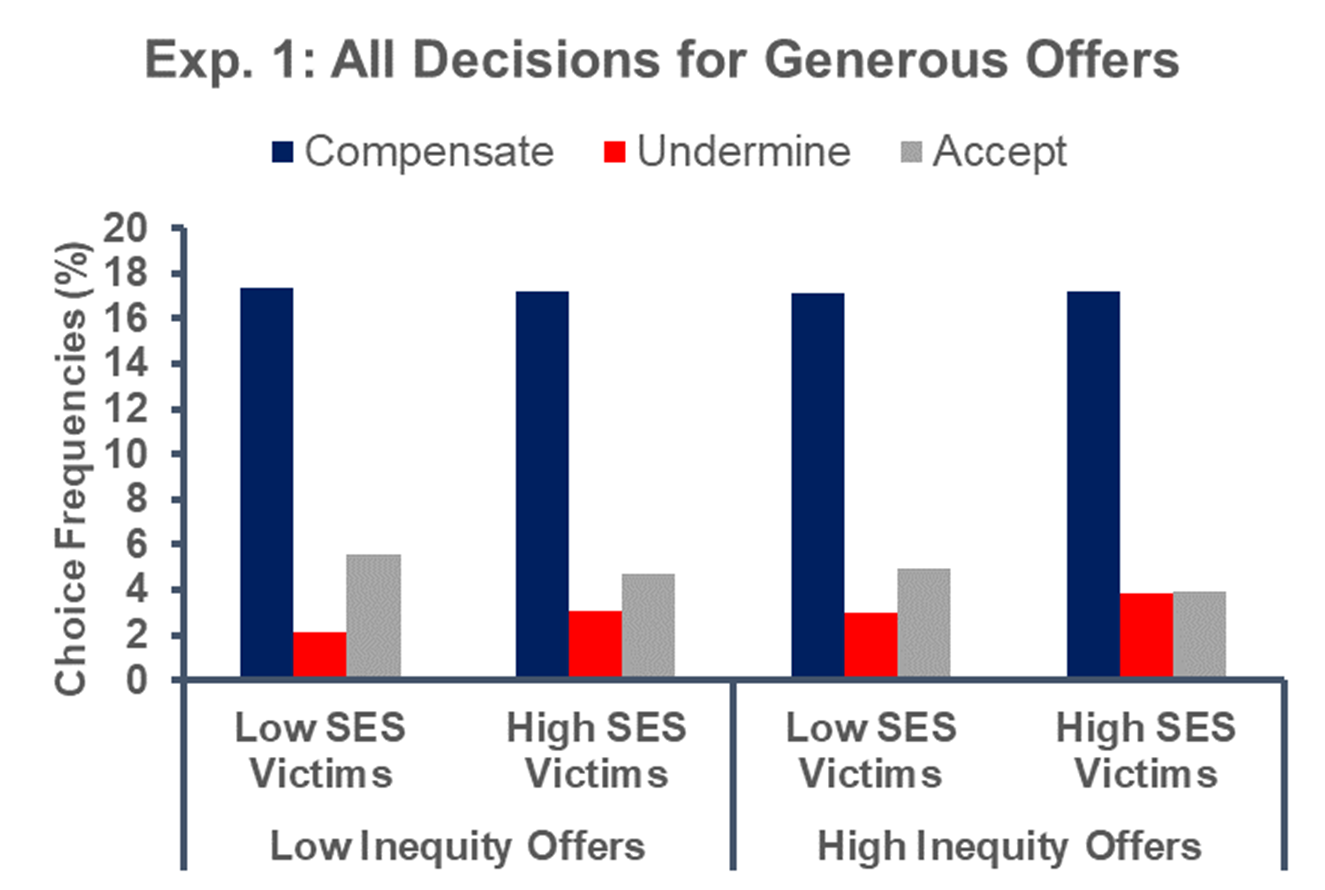


*Figure S8.* Percentage of choice frequencies (compensate, undermine, or accept) for hyper-generous offers are plotted as a function of offer inequity (i.e., low, high) and recipient SES (i.e., low, high). Analyses were conducted on count data, but percentages are plotted for ease of interpretation. For the undermine versus accept contrast, we observed independent main effects of offer inequity and recipient SES. For all contrasts with compensate, we observed significant SES × Inequity interactions. Due to multiple singular fits, we were unable to successfully model simple effects for any interaction.

**Compensate versus accept.** In this analysis, participants showed an overall preference to compensate over accept (see Figure S8), as indicated by a significant effect of the intercept, *b=*14.983, *SE=*0.749, *CI_95%_=*[13.516, 16.451], *z=*20.015, *p<*.001. We also observed significant main effects of recipient SES, *b=*2.315, *SE=*0.522, *CI_95%_=*[1.293, 3.338], *z=*4.439, *p*<.001, and offer inequity, *b=*1.558, *SE=*0.470, *CI_95%_=*[0.637, 2.478], *z=*3.315, *p=*.001, respectively. However, both effects were modulated by a significant SES × Inequity interaction, *b=*1.855, *SE=*0.521, *CI_95%_=*[0.833, 2.877], *z=*3.558, *p<*.001. Due to multiple singular fits, we were unable to successfully model simple effects for this interaction. However, an inspection of the means (see Figure S8) revealed that participants preferred to compensate over accept with increasing offer generosity. The magnitude of this generosity effect was 68.6% larger when the recipient was high SES than when the recipient was low SES.

**Undermine versus compensate.** In this analysis, we observed an overall preference to compensate over undermine (see Figure S8), indicated by a significant effect of the intercept, *b=-*16.356, *SE=*1.481, *CI_95%_=*[-19.259, -13.452], *z=*-11.041, *p<*.001. The main effects of recipient SES and offer inequity were both non-significant, *b=*0.150, *SE=*0.962, *CI_95%_=*[-1.736, 2.035], *z=*0.156, *p=*.876, and *b=*0.194, *SE=*0.964, *CI_95%_=*[-1.696, 2.084], *z=*0.201, *p=*.841, respectively. However, the SES × Inequity interaction was significant, *b=*1.649, *SE=*0.610, *CI_95%_=*[0.453, 2.845], *z=*2.703, *p=*.007. Due to multiple singular fits, we were unable to successfully model simple effects for this interaction. However, an inspection of the means (see Figure S8) revealed a greater preference to undermine over compensate with increasing offer generosity (but see non-significant main effect). The magnitude of this generosity effect was 40.0% larger when the recipient was low SES than when the recipient was high SES.

***Summary.*** As for selfish offers, participants also showed an overall preference for redistribution over the status quo even for patently hyper-generous offers. Unlike for selfish offers, participants who redistributed hyper-generous offers overwhelmingly preferred to compensate allocators at no cost to the recipient rather than undermining generosity by reversing the allocations. Nonetheless, we observed some variability in participants’ decisions to undermine (but not compensate) generous allocations. Participants increasingly preferred to undermine relative to the other two options (viz., accept, compensate) as a function of increasing recipient SES and offer inequity. In other words, participants were more reluctant to undermine generous allocations benefitting low-SES (vs. high-SES) recipients and allocators who made moderately (vs. very) hyper-generous offers.

## Experiment 2: Effects of Allocator SES on Redistribution Preferences

In Experiment 2, we manipulated the SES of the allocator (i.e., Player B), leaving the SES of the recipient (i.e., Player A) unspecified.

**Undermine versus accept.** In this analysis, participants showed an overall preference to undermine over accept (see Figure S9), indicated by a significant effect of the intercept, *b=*0.731, *SE=*0.198, *CI_95%_=*[0.343, 1.119], *z=*3.690, *p<*.001. We also observed a greater tendency to undermine over accept with increasing allocator SES (low SES: 53.5%; high SES: 67.0%) and offer inequity (low inequity: 56.8%; high inequity: 65.4%), as indicated by main effects of allocator SES, *b=*0.436, *SE=*0.155, *CI_95%_=*[0.132, 0.740], *z=*2.812, *p=*.005, and offer inequity, *b=*0.319, *SE=*0.125, *CI_95%_=*[0.074, 0.564], *z=*2.549, *p=*.011, respectively. Notably, the overall preference to undermine over accept was eliminated (i.e., both options were equally preferred) when the hyper-generous allocator’s SES was low, irrespective of offer inequity or when the offer was only moderately hyper-generous, irrespective of allocator SES (see Table S11 for pairwise comparisons). The SES × Inequity interaction was non-significant, *b=-*0.230, *SE=*0.124, *CI_95%_=*[-0.473, 0.013], *z=*-1.859, *p=*.063.


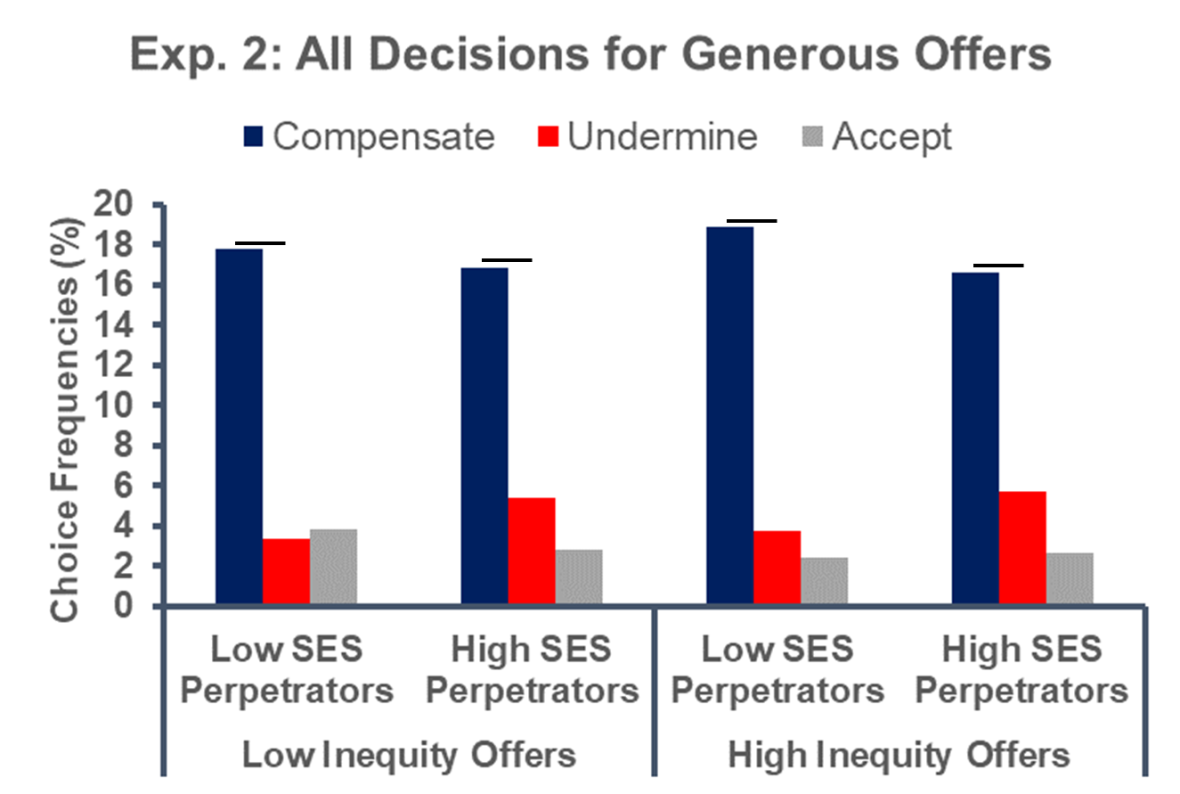


*Figure S9.* Percentage of choice frequencies (compensate, undermine, or accept) for hyper-generous offers are plotted as a function of offer inequity (i.e., low, high) and allocator SES (i.e., low, high). Analyses were conducted on count data, but percentages are plotted for ease of interpretation. Participants preferred to undermine (vs. accept) with increasing allocator SES and offer inequity. For undermine versus compensate contrast, we observed a significant SES × Inequity interaction. Significant simple differences for this interaction is indicated with horizontal bars. See Tables S11–S12 for contrast statistics from Experiment 2.

**Compensate versus accept.** In this analysis, participants showed an overall preference to compensate over accept (see Figure S9), indicated by a significant effect of the intercept, *b*=14.332, *SE=*0.782, *CI_95%_=*[12.799, 15.865], *z=*18.327, *p<*.001. All other effects were non-significant, *p*>.14.

**Undermine versus compensate.** In this analysis, participants showed an overall preference to compensate over undermine (see Figure S9), indicated by a significant effect of the intercept, *b=*-22.294, *SE=*1.189, *CI_95%_=*[-24.624, -19.965], *z=*-18.757, *p<*.001. We also observed significant main effects of allocator SES and offer inequity, *b=*4.009, *SE=*0.778, *CI_95%_=*[2.484, 5.533], *z=*5.153, *p<*.001, and *b=*7.837, *SE=*0.792, *CI_95%_=*[6.285, 9.389], *z=*9.896, *p<*.001, respectively. Both effects were modulated by a significant SES × Inequity interaction, *b=*-2.645, *SE=*0.635, *CI_95%_=*[-3.890, -1.401], *z=*-4.167, *p<*.001. Simple effects analyses revealed a greater preference to undermine over compensate with increasing offer generosity. The magnitude of this generosity effect was 106.4% larger when the allocator was high SES than when the allocator was low SES. See Table S12 for pairwise comparisons.

***Summary.*** Replicating Experiment 1, participants also showed an overall preference for redistribution over the status quo even for patently hyper-generous offers. Unlike for selfish offers, participants who redistributed hyper-generous offers overwhelmingly preferred to compensate allocators rather than undermining them by reversing their generous allocations. Nonetheless, we observed some variability in participants’ preference to undermine hyper-generous offers, thereby rewarding the allocator at the expense of the recipient. In the rare instances when participants chose to undermine rather than compensate (or accept) a hyper-generous allocator, this was more likely to occur for high-SES allocators. In the comparison with compensation, undermine decisions were especially likely when the high-SES allocator made a hyper-generous offer.

## Comparison of Recipient and Allocator SES Effects

To determine whether manipulating the recipient’s (vs. the allocator’s) SES differentially affected preferences to compensate or undermine hyper-generous allocations, we formally compared results across the experiments.

Results again revealed an overall preference to compensate over undermine (see Figure S10), indicated by a significant effect of the intercept, *b=*-15.000, *SE=*0.641, *CI_95%_=*[-16.256, -13.744], *z=*-23.410, *p<*.001. For both the recipient and the allocator, we observed significant main effects of SES and offer inequity, *b=*2.239, *SE=*0.430, *CI_95%_=*[1.396, 3.082], *z=*5.207, *p<*.001, and *b=*1.481, *SE=*0.394, *CI_95%_=*[0.708, 2.254], *z=*3.756, *p*<.001, respectively. Both effects were implicated in a significant SES × Inequity interaction, *b=*-1.733, *SE=*0.458, *CI_95%_=*[-2.630, -0.836], *z=*-3.787, *p*<.001. Simple effects analyses revealed a greater preference to undermine over compensate with increasing offer generosity. The magnitude of this generosity effect was 106.4% larger when the allocator was high SES than when the allocator was low SES. See Table S15 for pairwise comparisons. All effects of experiment were non-significant, *p*>.42.


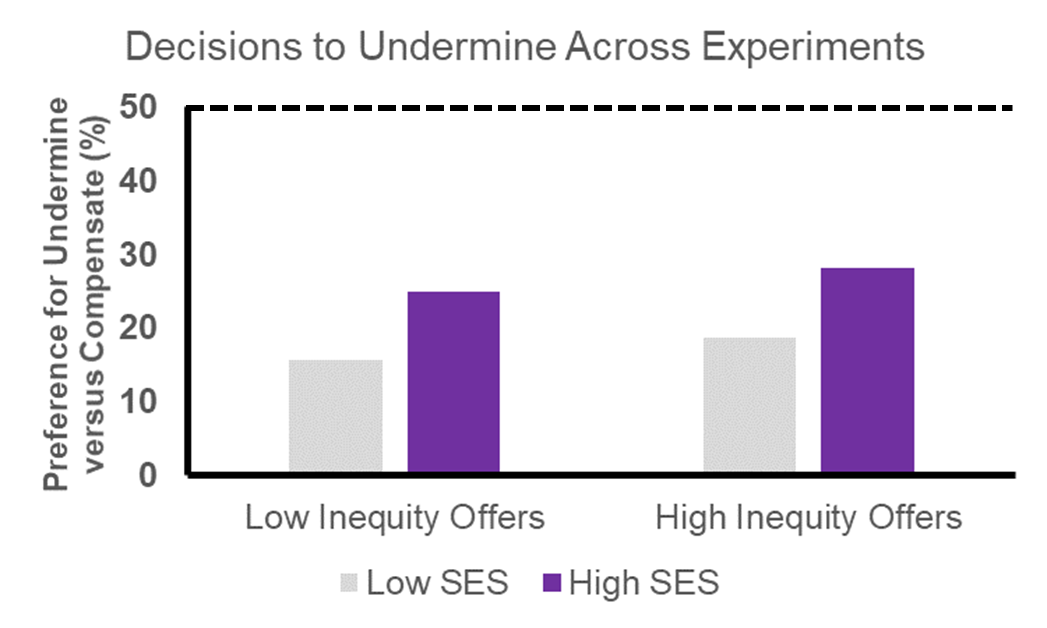


*Figure S10.* A formal analysis including data from both experiments indicated that redistribution preferences were shaped by both ascribed SES and offer inequity. Collapsing across experiment, preference for undermine (vs. compensate) for hyper-generous offers is plotted as a function SES and offer inequity. Analyses were conducted on count data, but percentages are plotted for ease of interpretation. All simple effects were non-significant (see Table S13). For all plotted conditions, participants preferred compensation to undermining generous offers (see Table S14).

## Discussion

As mentioned in the introduction to this section, hyper-generous offers provide a useful contrast to selfish offers in that only the former involve inequity without a clear victim. Somewhat surprisingly, participants showed a strong preference for redistribution over the status quo even when allocators made hyper-generous offers. Compared with the results for selfish offers reported in the main text and in Supplemental Text S2, our analyses of hyper-generous offers revealed qualitatively distinct SES- and inequity-based redistribution preferences. Unlike for selfish offers, participants overwhelmingly preferred to compensate hyper-generous allocators rather than accepting or undermining their allocations.

Although participants preferred to compensate rather than undermine in all conditions, we did observe some differences in the magnitude of this preference depending on offer generosity and the participants’ knowledge about SES. Unlike for our analyses of selfish offers (see main text and Supplemental Text S2), decisions to undermine versus compensate hyper-generous allocators did not reliably depend on whether we varied the SES of the recipient (Experiment 1) or the allocator (Experiment 2). In both experiments, we observed a relative reluctance of third parties to diminish the efficacy of generous offers that benefitted or were initiated by low-SES individuals, particularly when those offers were only moderately generous. At first blush, these findings appear to contrast with recent work by Hackel and Zaki that revealed a tendency to positively evaluate the generosity of high-status individuals over that of low-status individuals (Hackel & Zaki, 2018). In that study, high-status allocators had a larger pot of resources from which they could allocate. Importantly, when the amount of resources to be distributed is the same regardless of the allocator’s status as it was in the present study, it appears that participants may view generosity from low-status individuals as something to be encouraged through compensation, or at least not undermined.

In conclusion, our exploratory analyses of hyper-generous offers illustrate that even when people sacrifice for the benefit of others, third parties prefer to mitigate the resulting inequity. Overall, participants generally preferred to compensate hyper-generosity, consistent with the numerous perks afforded to philanthropists ranging from social capital to tax breaks. Complementing the finding that participants tended to punish allocators who victimized low-SES recipients, we found some evidence that participants favored low-SES recipients/allocators in the context of hyper-generous allocations. Specifically, these findings demonstrate a reluctance to undermine the welfare of low-SES beneficiaries and the efficacy of low-SES allocators. Findings also revealed a preference not to undermine moderately generous offers, irrespective of SES. Non-profits looking to optimize donations from small-scale donors from lower SES backgrounds may do well to consider these findings when crafting their appeals. Complementing the importance of not undermining low-SES donors, existing work also highlights the importance of mitigating perceptions of low-SES beneficiaries that they are pitied by their benefactors so as to avoid unintended negative emotions (Sandstrom, Schmader, Croft, & Kwok, 2019). Although such affect is perhaps not directly relevant to the third-party decision maker, it is nonetheless important to any low-SES beneficiary that the allocator and arbitrator may be attempting to help.
