# Supplementary material for: Punishing the privileged: Selfish offers from high-status allocators elicit greater punishment from third-party arbitrators
Source: PLoS One. 2020 May 14;15(5):e0232369. doi: 10.1371/journal.pone.0232369 (PMC7224526; doi:10.1371/journal.pone.0232369)
Supplement: S4 Text — (DOCX) [file pone.0232369.s004.docx]

# Supplemental Text S4: Exploratory Measures and Demographic Items

Following the Justice Game, participants completed several exploratory measures for use in future research and demographic items. Measures in this section are listed in their order of administration. Extended analyses from the main text that include subjective SES are reported in Supplemental Text S5.

## Subjective SES

First, participants completed the MacArthur ladder measure of subjective SES (Adler, Epel, Castellazzo, & Ickovics, 2000). This is a single-item measure of subjective status based on a picture of a ladder. Participants received the following instructions: “Think of this ladder as representing where people stand in the United States. At the top of the ladder (10^th^ step) are the people who are the best off: those who have the least money, least education, and the least respected jobs or no job. The higher up you are on this ladder, the closer you are to people at the very top; the further down you are, the closer you are to the people at the very bottom. Where would you place yourself on this ladder? Please select the step of the ladder you think you stand on at this time in your life, relative to other people in the United States.”

Prior to analyses of subjective SES, ratings were converted to Z scores. This was done separately for the datasets used in Experiments 1–2 and for the combined dataset.

## Objective SES

Following previous recommendations (Cloutier, Cardenas-Iniguez, Gyurovski, Barakzai, & Li, 2016), we administered a number of single-item measures that reflect distinct facets of SES. The items detailed below are grouped under three subcomponents of status: education, income, and current assets.

**Years of education.** What is the highest grade (or year) of regular school you have completed? Select one.

- Elementary School 01
- Elementary School 02
- Elementary School 03
- Elementary School 04
- Elementary School 05
- Elementary School 06
- Elementary School 07
- Elementary School 08
- High School 09
- High School 10
- High School 11
- High School 12
- College 13
- College 14
- College 15
- College 16
- Graduate School 17
- Graduate School 18
- Graduate School 19
- Graduate School 20+

**Education level.** What is the highest level of education you have completed?

- No formal education
- Elementary school
- Some high school
- Completed high school
- Some college
- BA/BS degree
- Some graduate/professional school
- Hold graduate/professional degree

**Personal income.** How much did you earn before taxes and other deductions during the past 12 months?

- Less than $5,000
- $5,000 through $11,999
- $12,000 through $15,999
- $16,000 through $24,999
- $25,000 through $34,999
- $35,000 through $49,999
- $50,000 through $74,999
- $75,000 through $99,999
- $100,000 and greater
- I don’t know
- No response

**Family income.** Which of these categories best describes your total combined family income for the past 12 months? This should include income (before taxes) from all sources, wages, rent from properties, social security, disability and/or veteran's benefits, unemployment benefits, workman's compensation, help from relatives (including child payments and alimony), and so on.

- Less than $5,000
- $5,000 through $11,999
- $12,000 through $15,999
- $16,000 through $24,999
- $25,000 through $34,999
- $35,000 through $49,999
- $50,000 through $74,999
- $75,000 through $99,999
- $100,000 and greater
- I don’t know
- No response

**Household savings viability.** If you lost all your current source(s) of household income (your paycheck, public assistance, or other forms of income), how long could you continue to live at your current address and standard of living?

- Less than 1 month
- 1 to 2 months
- 3 to 6 months
- 7 to 12 months
- More than 1 year

**Household savings amount.** Suppose you needed money quickly, and you cashed in all of your (and your spouse's) checking and savings accounts, and any stocks and bonds. If you added up what you would get, about how much would this amount to?

- Less than $500
- $500 to $4,999
- $5,000 through $9,999
- $10,000 through $19,999
- $20,000 through $49,999
- $50,000 through $99,999
- $100,000 through $199,999
- $200,000 through $499,999
- $500,000 and greater
- I don’t know
- No response

**Net worth.** If you now subtracted out any debt that you have (credit card debt, unpaid loans including car loans, home mortgage), about how much would you have left?

- Less than $500
- $500 to $4,999
- $5,000 through $9,999
- $10,000 through $19,999
- $20,000 through $49,999
- $50,000 through $99,999
- $100,000 through $199,999
- $200,000 through $499,999
- $500,000 and greater
- I don’t know
- No response

## Demographics

Next, participants responded to items asking about their age (entered as a number), gender (“male”, “female”, or “other”), ethnicity (“Black/African American”, “Asian/Asian American”, “White/Euro-American”, “Latino/Hispanic American”, “Middle Eastern/Arab American”, “Native American”, “Biracial/Multiracial”, “Other [please specify]”), political party (“Democrat”, “Republican”, “Other”), type of high school (“General public”, “Specialized or magnet public”, “Private”, “Parochial or religious”), and family social class (“Poor”, “Working Class”, “Middle Class”, “Upper Middle Class”, and “Upper Class”).

## Sociopolitical Preferences

Next, participants responded to items asking about their political views. This section consistent of three items.

**Political party preference.** How would you describe your political party preference?

- Strong Republican
- Weak Republican
- Independent Republican
- Independent
- Independent Democrat
- Weak Democrat
- Strong Democrat
- Other (please specify)

**Fiscal conservatism.** In terms of economic issues, how would you describe your political attitudes and beliefs?

- Very Conservative
- Conservative
- Slightly Conservative
- Middle-of-the-road
- Slightly Liberal
- Liberal
- Very Liberal
- Other (please specify)

**Social conservatism.** In terms of social issues, how would you describe your political attitudes and beliefs?

- Very Conservative
- Conservative
- Slightly Conservative
- Middle-of-the-road
- Slightly Liberal
- Liberal
- Very Liberal
- Other (please specify)

## National Identity

In a final block of questions, participants responded to items asking about their national origins and political orientation. Upon completion of these items, participants were debriefed and made arrangements to receive compensation via Amazon’s Mechanical Turk.

**Language.** What is your native language?

**Country of birth.** What country were you born in?

**Residency in the U.S.** If you were born in a country other than the United States, how long have you lived in the U.S.? If you were born in the United States, please enter N/A, and go on to the next question.

**Political orientation.** We hear a lot of talk these days about liberals and conservatives. Where on the following scale of political orientation would you place yourself?

- 1 - Extremely Liberal
- 2
- 3 - Moderately Liberal
- 4
- 5
- 6 - Neither
- 7
- 8
- 9 - Moderately Conservative
- 10
- 11 - Extremely Conservative
