# Supplementary material for: Punishing the privileged: Selfish offers from high-status allocators elicit greater punishment from third-party arbitrators
Source: PLoS One. 2020 May 14;15(5):e0232369. doi: 10.1371/journal.pone.0232369 (PMC7224526; doi:10.1371/journal.pone.0232369)
Supplement: S5 Text — (DOCX) [file pone.0232369.s005.docx]

# Supplemental Text S5: Standard Model Optimization Procedure

In the event of convergence failure and/or an over-fitted full model, we followed the standard procedure reported in this section. We followed this procedure for all analyses, including those reported in the main text. The random effects structures for all reported models can be viewed in the study analysis script available on the Open Science Framework at https://osf.io/9bxpk/?view_only=f29e16c77bf344c09067479d445d78a0.

1) Increase the maximum iterations allowed for convergence (up to 100,000) and reset the optimizer used for convergence.

2) Remove all correlation parameters from the random effects structure, run this model, and conduct a PCA on the resulting model to determine how many random effects can be supported by the data, as detailed by Bates and colleagues (Bates, Kliegl, Vasishth, & Baayan, 2015).

3) Based on the number of redundant dimensions in these data (determined in the previous step), remove slopes from the random effects structure, starting with random slopes corresponding to highest order interactions.  When choosing between random effects for interactions on the same order (e.g., two two-way interactions), remove the random slopes that account for the least amount of variance in the model from the previous step.

4) Re-run the PCA on the reduced model for step 3 to ensure that the model is not overfitted.  If the reduced model remains over-fitted (i.e., contains components that account for exactly zero variance), remove random slopes and repeat the PCA until all components account for some non-zero amount of variance.

5) Next, add in all possible correlation parameters, running PCA to avoid over-fitting the model.

a) If adding even one correlation parameter reduces the dimensionality of these data, then do not add any correlation parameters.  Use the final model from step 4.

b) If it is possible to include some but not all correlation parameters without reducing the dimensionality of these data, run all possible models with one correlation parameter excluded. The best model should converge and outperform all other models with one correlation parameter excluded as well as the final reduced model from step 4. Model comparison tests should be conducted using a log-likelihood ratio test.  (For more information about model comparison statistics: see [https://www.r-bloggers.com/how-do-i-interpret-the-aic/](https://www.google.com/url?q=https%3A%2F%2Fwww.r-bloggers.com%2Fhow-do-i-interpret-the-aic%2F&sa=D&sntz=1&usg=AFQjCNGchJPzxCgTDvaQHB54EmP5p0fIXg).) If necessary, exclude more than one correlation parameter and follow the same steps for running and comparing models. If no model with correlation parameters meets the selection criteria, then use the simpler model resulting from step 4.

c) If the dimensionality of these data is conserved after including all possible correlation parameters but the model with all possible correlation parameters results in a non-significant log-likelihood ratio test when compared with the final reduced model from step 4, then use the simpler model resulting from step 4.
