# Supplementary material for: Punishing the privileged: Selfish offers from high-status allocators elicit greater punishment from third-party arbitrators
Source: PLoS One. 2020 May 14;15(5):e0232369. doi: 10.1371/journal.pone.0232369 (PMC7224526; doi:10.1371/journal.pone.0232369)
Supplement: S6 Text — (DOCX) [file pone.0232369.s006.docx]

# Supplemental Text S6: Exploratory Analyses of Subjective SES

In the main text, we reported analyses showing that participants favored low-SES individuals in their decisions to punish allocators who made selfish offers. Our primary interpretations of these results centered on beliefs about the poor and the wealthy (e.g., stereotypes, norms). For example, the notion participants may use punishment as a means of correcting instances of extreme inequality reflects the expectation that the rich can and should be generous or at least non-exploitative in their treatment of the poor (Fiddick, Cummins, Janicki, Lee, & Erlich, 2013). However, an alternative explanation is that these effects of SES on punishment decisions could be explained by simple ingroup favoritism. In other words, participants may have preferred to favor low-SES individuals because they also identified as low SES—resulting in a shared group identity (Tajfel, 1982). To determine whether punishment decisions related to perceived SES were better explained by the perceiver’s own SES, we conducted exploratory analyses that included the perceiver’s subjective SES (Adler et al., 2000) in the models reported in the main text.

## Analysis Overview

In separate mixed-effect logistic regressions for each experiment, we tested how decisions to punish (punish = 1) versus not punish (compensate = 0, accept = 0) were predicted by offer inequity (extremely selfish = 1, moderately selfish = -1), recipient SES (high SES = 1, low SES = -1), subjective SES (continuous standardized predictor), and all possible interactions between these factors. To the extent possible, we allowed for between-participants variance in intercepts and slopes for all within-participant factors (i.e., random effects) and the correlations between these random effects. If the full model converged and was not over-fitted (as determined by the PCA procedure detailed in Supplemental Text S5), then we used the full model for our final model in line with existing recommendations (Barr, Levy, Scheepers, & Tily, 2013). However, sometimes, models failed to converge or were over-fitted (Bates, Kliegl, et al., 2015). In the event that the full models failed to converge or were over-fitted, we followed the steps outlined Supplemental Text S5.

## Results

**Experiment 1.** In the first experiment, we manipulated the ascribed SES of the recipient of the unfair offers (i.e., Player B) while the SES of the allocator (i.e., Player A) was left unspecified. As in the main text, we observed an overall preference not to punish relative to non-punitive options, as indicated by a significant effect of the intercept, *b=*-18.259, *SE=*0.894, *CI_95%_=*[-20.011, -16.507], *z=*-20.427, *p*<.001. We again observed a significant main effect of recipient SES, *b=*-3.285, *SE=*0.800, *CI_95%_=*[-4.854, -1.716], *z=*-4.104, *p*<.001, but not offer inequity, *b=*-0.632, *SE=*0.684, *CI_95%_=*[-1.973, 0.709], *z=*-0.924, *p*=.355. However, both effects were again implicated in a significant SES × Inequity interaction, *b=*-2.886, *SE=*0.348, *CI_95%_=*[-3.568, -2.204], *z=*-8.298, *p*<.001. The main effect of subjective SES (*p*=.507) and all interactions implicating this factor (*p* > .354) were non-significant.

With the exception of the main effect of offer fairness, the results from this analysis were consistent with those reported in the main text.

**Experiment 2.** In the second experiment, we manipulated the SES of the allocator (i.e., Player A) while the SES of the recipient (i.e., Player B) was left unspecified. As in the main text, we observed an overall preference not to punish relative to non-punitive options, as indicated by a significant effect of the intercept, *b=*-1.140, *SE=*0.142, *CI_95%_=*[-1.417, -0.862], *z=*-8.043, *p*<.001. Also as before, we observed significant main effects of recipient SES, *b=*0.625, *SE=*0.078, *CI_95%_=*[0.473, 0.777], *z=*8.057, *p*<.001, and offer inequity, *b=*0.945, *SE=*0.084, *CI_95%_=*[0.780, 1.110], *z=*11.211, *p*<.001. Unlike in the main text, the SES × Inequity interaction as non-significant, *b=*0.010, *SE=*0.066, *CI_95%_=*[-0.120, 0.139], *z=*0.144, *p*=.885. As for Experiment 1, the main effect of subjective SES (*p*=.075) and all interactions implicating this factor (*p* > .238) were non-significant.

With the exception of the SES × Inequity interaction, the results from this analysis were consistent with those reported in the main text.

**Comparison across experiments.** Although including subjective SES in the models led to results that were mostly similar to those reported in the main text, there were in both experiments some discrepancies. In Experiment 1, the main effect of offer fairness was eliminated. In Experiment 2, the SES × Inequity interaction was eliminated. These differences suggest that, despite null effects of subjective SES, the inclusion of this variable in the models was explaining some of the variance. Accordingly, we conducted one last exploratory model combining the data from both experiments, as in the main text. In other words, we modeled the preference for punishment (vs. non-punitive alternatives) as a function of perceived SES, offer inequity, experiment, and subjective SES.

As in the main text, the results revealed a prevailing preference for non-punitive options, as indicated by a significant effect of the intercept, *b=*-26.222, *SE=*0.734, *CI_95%_=*[-27.661, -24.784], *z=*-35.722, *p*<.001. Additionally, we again observed a greater tendency to punish financial offers were highly (vs. moderately) unfair, as indicated by a significant main effect of offer inequity, *b=*8.106, *SE=*0.302, *CI_95%_=*[7.513, 8.698], *z=*26.826, *p*<.001. Unlike in the main text, the main effects of perceived SES and experiment were non-significant, *b=*0.159, *SE=*0.223, *CI_95%_=*[-0.277, 0.596], *z=*0.716, *p*=.474, and *b=*0.037, *SE=*0.476, *CI_95%_=*[-0.896, 0.970], *z=*0.077, *p*=.939, respectively. However, the Perceived SES × Experiment interaction reported in the main text remained significant, *b=*7.650, *SE=*0.301, *CI_95%_=*[7.059, 8.241], *z=*25.378, *p*<.001. As a reminder, this interaction was characterized by greater punishment for perpetrators with presumed higher standing relative to the victim. As depicted in Figure 2 in the main text, we observed an increased preference to punish selfish allocators as a function of decreasing recipient SES (Experiment 1) and increasing allocator SES (Experiment 2). Critically, this two-way interaction was further modulated by offer inequity (three-way interaction: *b=*-1.050, *SE=*0.197, *CI_95%_=*[-1.436, -0.663], *z=*-5.317, *p*<.001) and both offer inequity and subjective SES (four-way interaction: *b=*0.432, *SE=*0.203, *CI_95%_=*[0.033, 0.830], *z=*2.124, *p*=.034). All other effects were non-significant, *p*>.312.

To follow up on the four-way interaction, we assessed the reliability of the Perceived SES × Offer Inequity × Experiment interaction at low (-1 SD), mean, and high (+1 SD) subjective SES. For illustrative purposes, we also plotted the sample’s distribution of subjective SES scores (Figure S11) and preferences for punishment according to three discrete segments of our sample (Figure S12): low SES (1–4 on the MacArthur scale, *n*=433), middle SES (5–6 on the MacArthur scale, *n*=397), and high SES (7–10 on the MacArthur scale, *n*=169).


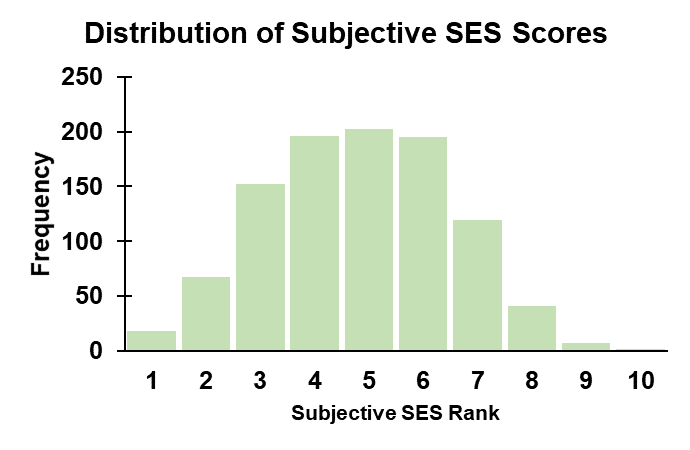


*Figure S11.* Distribution of subjective SES scores in the combined sample from Experiments 1 and 2. Scores were centered below the scale’s midpoint but were otherwise normally distributed.


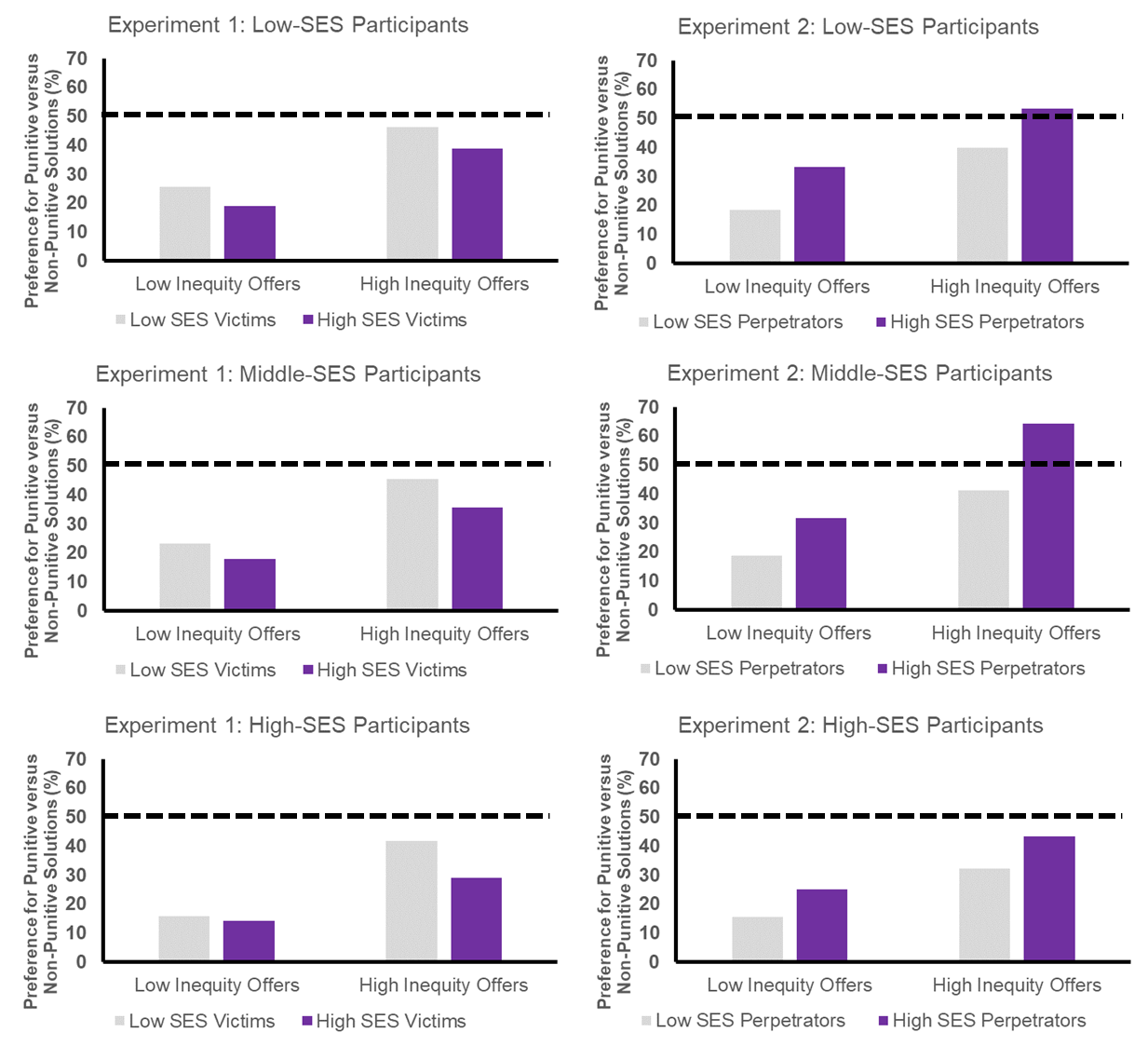


*Figure S12.* Depiction of SES × Offer Inequity × Experiment interaction for discrete groups based on subjective SES: low (top two panels, ranks 1–4, *n*=433), middle (center two panels, ranks 5–6, *n*=397), and high (bottom two panels, ranks 7–10, *n*=169). The horizontal dashed bar represents equal preference for punitive versus all non-punitive options. These plots were not generated as part of any analysis and are intended for solely illustrative purposes. Therefore, we do not indicate tests of simple effects in this figure.

***Low-SES participants.*** At -1 SD (approximately a 3 on the MacArthur scale), the Perceived SES × Offer Inequity × Experiment interaction was significant, *b=*-1.481, *SE=*0.304, *CI_95%_=*[-2.078, -0.885], *z=*-4.870, *p*<.001. To further decompose this interaction, we next tested for Perceived SES × Offer Inequity interactions separately by experiment.

For Experiment 1, the Perceived SES × Offer Inequity interaction was significant, *b=*1.431, *SE=*0.452, *CI_95%_=*[0.546, 2.316], *z=*3.169, *p*=.002. Simple effects indicated that participants increasingly preferred punitive solutions as a function of decreasing recipient SES, but only for highly unfair offers, *b=*-0.369, *SE=*0.141, *CI_95%_=*[-0.645, -0.094], *z=*-2.625, *p*=.009, and not moderately unfair offers, *b=*-0.518, *SE=*0.538, *CI_95%_=*[-1.571, 0.536], *z=*-0.963, *p*=.335.

For Experiment 2, the Perceived SES × Offer Inequity interaction was significant, *b=-*1.434, *SE=*0.379, *CI_95%_=*[-2.176, -0.691], *z=*-3.785, *p*<.001. Simple effects indicated that participants increasingly preferred punitive solutions as a function of increasing allocator SES, but only for highly unfair offers, *b=*0.623, *SE=*0.129, *CI_95%_=*[0.370, 0.875], *z=*4.840, *p*<.001, and not moderately unfair offers, *b=*0.966, *SE=*0.548, *CI_95%_=*[-0.108, 2.040], *z=*1.763, *p*=.078.

***Mean-SES participants*.** At mean SES (approximately a 5 on the MacArthur scale), the Perceived SES × Offer Inequity × Experiment interaction was significant, *b=*-1.050, *SE=*0.197, *CI_95%_=*[-1.436, -0.663], *z=*-5.317, *p*<.001. To further decompose this interaction, we next tested for Perceived SES × Offer Inequity interactions separately by experiment.

For Experiment 1, the Perceived SES × Offer Inequity interaction was significant, *b=*0.834, *SE=*0.299, *CI_95%_=*[0.247, 1.420], *z=*2.787, *p*=.005. Simple effects indicated that participants increasingly preferred punitive solutions as a function of decreasing recipient SES, but only for highly unfair offers, *b=*-0.447, *SE=*0.104, *CI_95%_=*[-0.652, -0.243], *z=*-4.287, *p*<.001, and not moderately unfair offers, *b=*-0.333, *SE=*0.398, *CI_95%_=*[-1.113, 0.447], *z=*-0.837, *p*=.403.

For Experiment 2, the Perceived SES × Offer Inequity interaction was non-significant, *b=*0.010, *SE=*0.066, *CI_95%_=*[-0.120, 0.140], *z=*0.150, *p*=.881.

***High-SES participants*.** At +1 SD (approximately a 7 on the MacArthur scale), the Perceived SES × Offer Inequity × Experiment interaction was non-significant, *b=*0.120, *SE=*0.072, *CI_95%_=*[-0.021, 0.261], *z=*1.668, *p*=.095. Instead, we observed a significant Perceived SES × Experiment interaction, *b=*0.451, *SE=*0.074, *CI_95%_=*[0.306, 0.597], *z=*6.086, *p*<.001. As in the omnibus model above and in the final combined analysis reported in the main text, this interaction was characterized by greater punishment of allocators who exploited low-SES (vs. high-SES) victims in Experiment 1 and of high-SES (vs. low-SES) allocators in Experiment 2 (see Figure S12).

## Discussion

Overall, results from these supplemental analyses were largely consistent with those reported in the main text, where the participant’s subjective SES was not included in the model. However, we found some evidence that the perceiver’s SES was accounting for variance in the data, though not in a manner consistent with ingroup favoritism. It seems that low-SES participants were indeed more sensitive to distinct combinations of perceived SES, offer fairness, and whether the recipient’s or allocator’s SES was manipulated. Low-SES participants showed the most pronounced tendency to increase their punishment of (1) allocators who made very unfair offers to low-SES (vs. high-SES) recipients and (2) high-SES (vs. low-SES) allocators who made very unfair offers. However, even high-SES participants showed a pattern of results that looked far more like that of low-SES participants than would be expected if they were favoring their own ingroup. If ingroup favoritism were wholly at play, one might expect a greater tendency for high-SES participants to favor the advantaged party in these financial allocations. Instead, high-SES participants actually favored the disadvantaged when deciding whether to punish allocators. Although this preference was perhaps not as strong as for low-SES participants, the results are enough to suggest that the results reported in the main text are not exclusively due to ingroup favoritism. More consistent with our favored belief-based interpretation of the results, this final set of analyses suggest that participants across the spectrum of SES favored the individual perceived to be relatively disadvantaged, albeit in different ways. The pattern of means displayed in Figure S12 suggests greater concern about the exploitation of low-SES victims among high-SES participants and greater concern about high-SES perpetrators among low- to mid-SES participants. Further work is needed to explore the role of the perceiver’s own status in the use of punishment as a form of addressing financial inequity.

# References

Adler, N. E., Epel, E. S., Castellazzo, G., & Ickovics, J. R. (2000). Relationship of subjective and objective social status with psychological and physiological functioning: Preliminary data in healthy white women. *Health Psychology*, *19*, 586–592. https://doi.org/10.1037/0278-6133.19.6.586

Barr, D. J., Levy, R., Scheepers, C., & Tily, H. J. (2013). Random effects structure for confirmatory hypothesis testing: Keep it maximal. *Journal of Memory and Language*, *68*, 255–278. https://doi.org/10.1016/j.jml.2012.11.001

Bates, D., Kliegl, R., Vasishth, S., & Baayan, H. (2015). *Parsimonious mixed models*. Retrieved from https://arxiv.org/pdf/1506.04967v1.pdf

Bates, D., Maechler, B., Bolker, B., & Walker, S. (2015). Fitting linear mixed-effects models using lme4. *Journal of Statistical Software*, *67*, 1–48. https://doi.org/10.18637/jss.v067.i01

Bekkers, R., & Wiepking, P. (2011). A literature review of empirical studies on philanthropy: Eight mechanisms that drive charitable giving. *Nonprofit and Voluntary Sector Quarterly*, *40*, 924–973. https://doi.org/10.1177/0899764010380927

Cloutier, J., Cardenas-Iniguez, C., Gyurovski, I., Barakzai, A., & Li, T. (2016). Neuroimaging investigations of social status and social hierarchies. In J. R. Absher & J. Cloutier (Eds.), *Neuroimaging personality, social cognition, and character* (pp. 187–203). https://doi.org/10.1016/B978-0-12-800935-2.00009-9

FeldmanHall, O., Sokol-Hessner, P., Van Bavel, J. J., & Phelps, E. A. (2014). Fairness violations elicit greater punishment on behalf of another than for oneself. *Nature Communications*, *5*(5306). https://doi.org/10.1038/ncomms6306

Fiddick, L., Cummins, D. D., Janicki, M., Lee, S., & Erlich, N. (2013). A cross-cultural study of noblesse oblige in economic decision-making. *Human Nature*, *24*, 318–335. https://doi.org/10.1007/s12110-013-9169-9

Hackel, L. M., & Zaki, J. (2018). Propagation of economic inequality through reciprocity and reputation. *Psychological Science*, *29*, 604–613. https://doi.org/10.1177/0956797617741720

R Core Team. (2019). *R: A language and environment for statistical computing*. Retrieved from https://www.r-project.org/

Sandstrom, G. M., Schmader, T., Croft, A., & Kwok, N. (2019). A social identity threat perspective on being the target of generosity from a higher status other. *Journal of Experimental Social Psychology*, *82*, 98–114. https://doi.org/10.1016/j.jesp.2018.12.004

Tajfel, H. (1982). Social psychology of intergroup relations. *Annual Review of Psychology*, *33*, 1–39. https://doi.org/10.1146/annurev.ps.33.020182.000245
